# Comparison of clinical efficacy of different acupuncture methods in the treatment of fibromyalgia: a network meta-analysis based on randomized controlled trials

**DOI:** 10.3389/fneur.2026.1869198

**Published:** 2026-08-05

**Authors:** Shanxi Wang, Haitao Liu, Lirong Zeng, Songtao Liu, Wei Tang

**Affiliations:** 1 Department of Rehabilitation Medicine, Affiliated Hospital (Clinical College) of Xiangnan University, Xiangnan University, Chenzhou, Hunan, China; 2 College of Medical Imaging Laboratory and Rehabilitation, Xiangnan University, Chenzhou, Hunan, China; 3 Department of Rehabilitation Medicine, The First People’s Hospital of Chenzhou (The First Affliated Hospital of Xiangnan University), Chenzhou, Hunan, China

**Keywords:** acupuncture, electroacupuncture, fibromyalgia, floating needle, network meta-analysis, randomized controlled trial

## Abstract

**Background:**

Fibromyalgia is a complex chronic pain syndrome for which current pharmacological treatments have limitations. Acupuncture, as a complementary therapy, has shown potential, but the efficacy differences among various acupuncture methods remain unclear.

**Methods:**

A network meta-analysis was conducted by systematically searching commonly used databases up to December 28, 2025, to identify relevant randomized controlled trials evaluating different acupuncture approaches for fibromyalgia. The primary outcome measure was visual analog scale (VAS), with total effective rate (TER) as a secondary exploratory indicator.

**Results:**

A total of 26 studies involving 2,037 patients were included. Pairwise meta-analysis showed that acupuncture combined with cupping and medication was superior to medication alone in improving VAS [WMD = −3.81, 95% CI (−6.04, −1.58)] and HAMD scores [WMD = −4.90, 95% CI (−6.15, −3.65)]. Regarding pain improvement (VAS score), a widely recognized core outcome measure internationally, electroacupuncture had the highest SUCRA value (0.99), indicating the highest probability of ranking first within this network meta-analysis. However, it should be noted that this ranking is based on limited evidence (only one small-sample study), resulting in very low certainty of evidence, and thus should be interpreted cautiously. For overall effective rate (TER, a composite endpoint commonly used in Chinese acupuncture trials), floating needle combined with cupping had the highest SUCRA value (0.74). Meta-regression suggested a positive correlation between acupuncture efficacy and treatment duration. CINeMA assessment of evidence quality indicated that the overall quality of evidence ranged from low to very low.

**Conclusion:**

Based on current low-quality evidence, the findings of this study should be regarded as exploratory. The SUCRA reflects probabilistic ranking rather than absolute clinical superiority, and these results require further validation in higher-quality studies.

## 1 Introduction

Fibromyalgia syndrome (FMS) is a complex chronic pain syndrome. Fibromyalgia has been recognized as the third most common musculoskeletal disorder, and its prevalence increases significantly with age (1). The global prevalence in the general population ranges from 0.2% to 6.6%, and the prevalence is even as high as 3.69% in Europe (2). Female patients with primary FMS syndrome in adolescents account for up to 95% (3). From the perspective of clinical manifestations, the disease is characterized by generalized musculoskeletal pain as the core feature, accompanied by a complex syndrome of fatigue, sleep disorders and multiple functional symptoms, leading to a serious decline in the quality of life of patients (4).

The treatment of FMS mainly relies on multimodal strategies, among which drug therapy plays a central role. Although duloxetine, milnacipran and pregabalin approved by the US FDA can relieve the pain of some patients, about 30% of patients do not respond to the treatment, and about 40% of patients stop treatment due to adverse reactions (such as nausea and insomnia) (5, 6). Non-pharmacological treatments such as transcranial direct current stimulation (tDCS) and photobiomdulation therapy have shown new therapeutic prospects, but their clinical significance still needs to be verified (7). As a non-invasive and drug-free option, low-intensity laser therapy can significantly reduce inflammatory markers and improve pain scores, but existing studies are mostly limited to short-term observation (8). Ozone therapy has been shown in small studies to improve symptoms by ≥50% in 70% of patients without serious adverse reactions, but its mechanism of action remains to be elucidated (9). Although safe, these alternative therapies lack large-scale, long-term follow-up data, and treatment standardization is insufficient. The main challenges of the current treatment system include: the individual variation of drug efficacy is significant, and about 40% of patients discontinue treatment because of adverse reactions (such as nausea and insomnia caused by duloxetine). The average period of existing clinical studies is only 10 weeks, which makes it difficult to evaluate the long-term efficacy (10). These limitations have prompted researchers to explore complementary and alternative therapies, among which acupuncture has attracted much attention due to its good safety and multi-target modulation potential. A combination therapy for multiple symptoms has not been established. Clinical practice guidelines emphasize the need to develop individualized strategies according to the patient’s specific symptom spectrum (such as pain-dominant or sleep-disturbance dominant) (11), but the lack of accurate classification biomarkers makes this goal still face significant obstacles.

The core pathological mechanism of FMS is considered to be central sensitization, which is the amplified processing of pain signals by the central nervous system. Studies have shown that FMS patients have multiple pathological changes such as descending pain inhibition pathway dysfunction, enhanced neuroinflammatory response, and HPA axis dysfunction (12). At the neuroimaging level, FMS patients show abnormal activation of pain processing brain areas (such as insula, anterior cingulate cortex and thalamus), and the functional connectivity patterns of default mode network and salience network are also changed. In addition, a variety of potential biomarkers have been reported, including neurotrophins, inflammatory cytokines and microglia markers in serum and cerebrospinal fluid (12). These mechanisms provide an important theoretical basis for understanding the targets of non-drug therapies such as acupuncture and moxibustion. In recent years, as a complementary treatment for FMS, acupuncture and moxibustion has attracted more and more attention in clinical application and scientific research. A number of systematic reviews and meta-analyses have suggested that acupuncture can improve pain, sleep disorders and quality of life in patients with FMS (13). However, there are several controversies in the existing evidence: there are still differences in the effect of acupuncture on fatigue symptoms (14); About 20%–30% of the patients of acupuncture and moxibustion has no reaction, the forecast factors needs to be clear (15); Moreover, the follow-up period of most studies is short, and the evidence of long-term efficacy is insufficient (15). The effect of acupuncture and moxibustion on improving fatigue symptoms is divergent, and some studies have failed to confirm its significant effect (16). About 20%–30% of the patients response to acupuncture treatment without forecast factors include high pressure points, pain disaster tendency, and at the same time use duloxetine (17). In terms of safety, acupuncture is well tolerated, and serious adverse events are rare. However, the inconsistent reporting standards of adverse events among different studies affect the reliability of safety assessment (18).

Although existing studies have confirmed the significant effects of acupuncture and moxibustion in improving pain, sleep disturbance and quality of life in patients with FMS, there are still key challenges in clinical practice: the relative efficacy differences between different acupuncture and moxibustion therapies are not clear, and the existing randomized controlled trials (RCTs) are significantly heterogeneous in terms of intervention regimens, evaluation indicators and follow-up periods. Traditional meta-analysis methods are difficult to directly compare a variety of acupuncture and moxibustion therapies without head-to-head trials, while network meta-analysis (NMA) can integrate direct and indirect comparative evidence at the same time by constructing an evidence network, so as to provide a more comprehensive order of efficacy for clinical decision-making. Especially for FMS, which is a complex disease involving multiple systems, it is necessary to systematically evaluate the differential efficacy of different acupuncture and moxibustion regimens on the core symptom clusters, as well as the safety and cost-effectiveness of long-term treatment. By integrating the RCT data from all over the world, this study will fill the existing evidence gap, provide evidence-based basis for the development of individualized acupuncture and moxibustion treatment plan, and ultimately achieve the goal of precision medicine.

## 2 Methods

### 2.1 Register

The study was conducted in compliance with the requirements of PRISMA-NMA. We have been registered with PROSPERO (ID: CRD420251252462).

### 2.2 Inclusion criteria

*Population*: Patients diagnosed with fibromyalgia syndrome were included in the study. The diagnostic criteria primarily followed those proposed by the American College of Rheumatology (ACR) (19). After reviewing the 26 included studies, the use of diagnostic criteria was as follows: 22 studies (84.6%) used the ACR 1990 criteria (requiring widespread pain lasting more than 3 months and tenderness in at least 11 of 18 specific tender points), 2 studies used the ACR 2010 criteria (Widespread Pain Index combined with the Symptom Severity Scale), and 2 studies used the ACR 2016 criteria. There were no restrictions regarding nationality, race, age, or gender.

*Interventions*: The included acupuncture and moxibustion interventions were divided into the following two categories: (a) simple acupuncture and moxibustion (including acupuncture, electroacupuncture, intradermal acupuncture, Fu’s acupuncture, needle-knife, moxibustion, acupoint thread-embedding and acupoint injection, etc.), and the key parameters such as acupuncture frequency (times/week), total treatment course (weeks) and duration of single treatment were reported. (b) acupuncture combined with other treatment methods (including combined medication, physical therapy, etc.). It should be emphasized that the definition of network nodes is based on the complete intervention protocols actually implemented in each study. When comparing combined intervention nodes with single intervention nodes within the same network, the effect size reflects the overall efficacy of the full intervention regimen, rather than the independent contribution of individual intervention components.

*Control group*: drug therapy (amitriptyline, tramadol, etc.) or acupuncture. If acupuncture was used in both the trial and control groups, it must be a different type of acupuncture.

*Outcome measures*: This study prespecifies three outcome measures, ranked by priority as follows:

1. Visual Analogue Scale (VAS) (20): As the core measure for assessing pain intensity, VAS is one of the six core outcome domains recommended by OMERACT and widely used in international clinical trials. VAS serves as the primary outcome measure in this study.
2. Hamilton Depression Rating Scale (HAMD) (21): Used to evaluate the common comorbid depressive symptoms in fibromyalgia patients. HAMD is the pre-specified secondary outcome measure in this study. Data extraction prioritizes post-treatment scores; for studies reporting only changes between pre- and post-treatment, the post-treatment mean is estimated based on available baseline data when possible. The network meta-analysis method for HAMD is consistent with that for VAS (see Section 2.7), using a frequentist framework, with continuous variables expressed as mean difference (MD) and 95% confidence interval (CI).
3. Total effective rate (TER): A composite endpoint commonly used in acupuncture clinical trials in China, which integrates assessments of tender point count, VAS, and HAMD scores. Criteria for judgment are as follows: cured (complete disappearance of body pain and tender points, HAMD score <17); markedly effective (≥50% reduction in number of tender points, HAMD, and VAS scores); effective (≥25% reduction in number of tender points, HAMD, and VAS scores); ineffective (<25% reduction in number of tender points, HAMD, and VAS scores). TER = (number of effective cases [cured + markedly effective + effective]) / total number of participants × 100%. It should be noted that TER has not yet been standardized or adopted internationally in fibromyalgia clinical trials; therefore, it is included here solely as an exploratory supplementary measure. For studies reporting both VAS and TER, VAS is prioritized for analysis; if only TER is reported, it will be included in the TER analysis. Since TER is not part of the OMERACT-recommended core outcome domains, the main conclusions of this study should be based on VAS results, with TER and HAMD findings serving as supplementary references.

### 2.3 Exclusion criteria

Duplicate publications; Non-compliance with interventions; Non-clinical multi-arm RCTS and CCTS, such as case reports, theoretical discussions, literature research, self-control, animal studies, etc. Unclear efficacy evaluation, no baseline data evaluation, different baseline treatment measures, no primary outcome indicators, and causal relationship determination of the final efficacy because of involving other treatment measures; Incomplete data, unknown study results, and data errors; The full text could not be obtained; A network meta-analysis could not be performed.

### 2.4 Literature search strategy

The following Chinese and English databases were searched by computer: PubMed, Web of science, Cochrane Library, Embase, China national knowledge infrastructure (CNKI), Wanfang data and VIP were searched database, SionMed, and Chinese databases were searched for randomized controlled trials (RCTS) of acupuncture and moxibustion for FMS. The search time limit was from the establishment of the database to December 28, 2025. A combination of subject headings and free words was used to search the database, and references of relevant articles were manually searched (Supplementary material 1).

### 2.5 Literature screening and data extraction

Two researchers independently screened the literature and extracted the data in strict accordance with the inclusion and exclusion criteria, and cross-checked them. If there was any disagreement, the discussion or consultation with a third party was conducted, and consensus was reached. The extracted contents included the first author, publication year, sample size, intervention and control measures, intervention time, main evaluation tools, and outcome indicators.

For VAS scores of continuous variables, this study adopted the following standardization strategies to enhance data comparability: (1) Scale range harmonization: The mean and standard deviation of 0–10 point VAS scores were multiplied by 10, uniformly converted to a 0–100 mm scale before pooling for analysis, thereby eliminating the confounding effect of different scale ranges on effect size measurement; (2) Consistent follow-up timing: Primary analyses were conducted using the first assessment time point after treatment completion (i.e., immediate post-treatment evaluation). For studies reporting multiple follow-up time points, the primary endpoint defined by the researchers was prioritized; if not explicitly defined, the most recent assessment after treatment completion was selected. Since all VAS data have been standardized to the same scale range (0–100 mm), this study used MD (mean difference) as the effect size measure, ensuring clinically interpretable results.

### 2.6 Risk of bias assessment

The risk of bias of the included studies was assessed using the ROB2 tool recommended by the Cochrane Handbook (22). ROB2 covers five domains: bias arising from the randomization process, bias due to deviations from intended interventions, bias due to missing outcome data, bias in measurement of the outcome, and bias in selection of the reported result. The risk of bias in each domain was classified into three levels: low risk, some concerns, and high risk. Two researchers independently conducted the assessment, compared the results, and consulted a third researcher or reached a consensus through discussion in case of disagreement.

### 2.7 Statistical analysis

#### 2.7.1 Pairwise meta-analysis

When the number of original studies directly comparing interventions was ≥3, a pairwise meta-analysis using a random-effects model was performed. Effect sizes were pooled using mean differences (MD), with 95% confidence intervals (CI) calculated. Heterogeneity was assessed using the I^2^ statistic: I^2^ > 50% was defined as high heterogeneity. If the number of included studies was 10 or more, publication bias was comprehensively evaluated by assessing funnel plot symmetry and Egger’s regression test.

#### 2.7.2 Network meta-analysis

The frequency framework was used for network meta-analysis. The statistical analysis was performed using the network package of Stata 18.0 and the netmeta package of R 4.3.2. relative risk (RR) was used for binary variables and mean difference (MD) was used for continuous variables in the outcome indicators, and the corresponding 95% confidence interval (CI) was calculated. CochraneQ test and I^2^ test were used for heterogeneity test. If *p* > 0.1 and *I*^2^ < 50%, the heterogeneity between studies was small, and the fixed effect model was used for analysis. If *p* < 0.1 or I^2^ ≥ 50%, the heterogeneity between studies was considered to be large, and the random effect model was used. First, the network evidence diagram was drawn to describe the direct comparison between the interventions. Effect sizes were pooled using a multivariate random-effects model based on restricted maximum likelihood estimation, and heterogeneity was assessed by Q test and I^2^ statistic (fixed-effects model when I^2^ < 50% and *p* > 0.10, random-effects model otherwise). The global consistency was assessed by design-treatment interaction model, and the local consistency was tested by node splitting method. The efficacy ranking of each intervention was quantified by the area under the cumulative ranking probability map [Surface Under the Cumulative Ranking Curve (SUCRA)]. The closer the SUCRA value is to 1, the higher the probability that the intervention ranks first. Sensitivity analysis was performed by excluding studies one by one or by including only randomized controlled trials. Publication bias was assessed using a comparative-corrected funnel plot, supplemented by Egger regression test when necessary. Meta regression method was used to explore the influence of treatment course, acupuncture frequency and other factors on the curative effect. The specific method was to fit the regression model of acupuncture and drug treatment under the framework of linear regression, taking the treatment course as a covariate, and to evaluate the correlation between the treatment course and the effect size and its statistical significance.

### 2.8 Quality of evidence evaluation

We evaluated the quality of evidence using CINeMA (23). CINeMA contains six domains: within-study bias, between-study bias, indirectness, imprecision, heterogeneity and inconsistency to grade the quality of evidence. Finally, the quality of evidence was divided into four levels: high, medium, low and very low quality.

## 3 Results

### 3.1 Literature screening process

**Figure 1:**
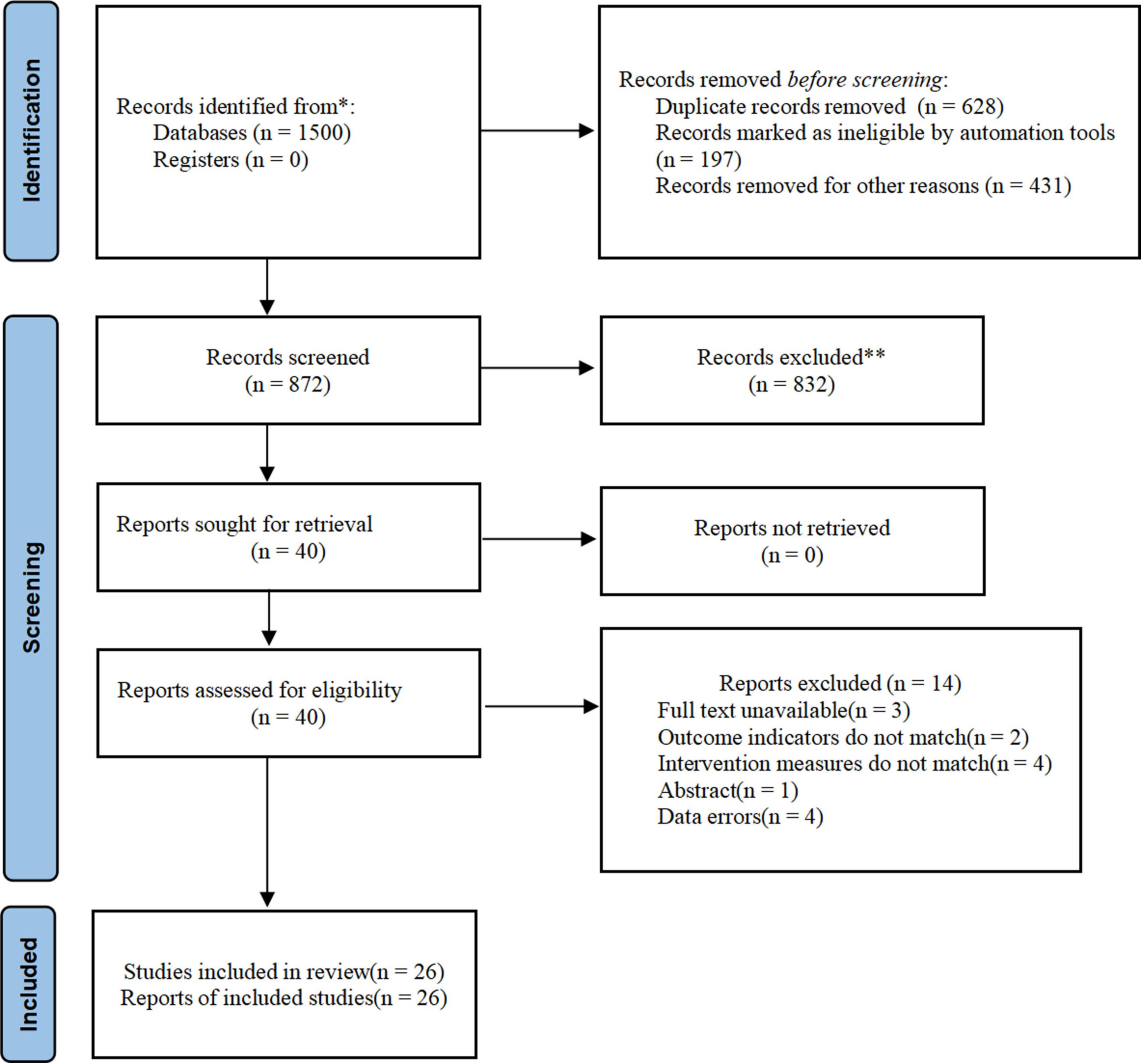
Flowchart of literature screening process (PRISMA flow diagram) [*Consider, if feasible to do so, reporting the number of records identified from each database or register searched (rather than the total number across all databases/registers). **If automation tools were used, indicate how many records were excluded by a human and how many were excluded by automation tools].

After strict screening, 14 articles were excluded from the full text (3 articles could not be obtained, 2 articles had inconsistent outcome indicators, 4 articles had inconsistent intervention measures, 1 was an abstract, and 4 articles had data errors). Finally, 26 studies were included (Figure 1).

### 3.2 Basic characteristics of the studies

The 26 articles included in this study included 2 three-arm clinical trials and 24 two-arm randomized controlled trials (24–49). A total of 2,037 patients were included. The earliest publication was in 1992 and the most recent in 2024. Published countries are from China, Brazil, Sweden, and Iran. Acupuncture methods include acupuncture (A), acupoint thread embedding therapy (ATET), thumb tack needle (TTN), medicine-separated moxibustion (MSM), acupuncture and moxibustion. Acupoint injection (AI), Fu’s Subcutaneous needling (FSN), thermosensitive moxibustion (TM), small needle-knife (SNF), fire needle(FN), electropuncture (EA) (Table 1).

**Table 1:** Basic characteristics of included studies.

Author	Year	Country	Intervention		Frequency	Samplesize		Sex(M/WM)		Age		Time	Outcomes
			I	C		I	C	I	C	I	C		
Cao and Li (23)	2003	China	A + C + M	M	Once every three days	28	28	12/44		42.1 ± 14.5		4w	TER, HAMD
Chen et al. (24)	2024	China	A + TCM	M	Once a day	30	30	17/13	14/16	45.47 ± 7.8	43.17 ± 7.91	4w	VAS
Gong and Wang (25)	2010	China	A	M	Once a day	30	30	9/21	11/19	35 ± 8	34 ± 6	12w	VAS, TER, HAMD
Guo et al. (26)	2014	China	ATET	M	Once every ten days	30	30	6/24	8/22	33.2 ± 9.75	32.4 ± 10.43	4w	VAS, TER
Jiang et al. (27)	2010	China	A + C + M/A + C/M		Once a day	62/64/60		19/43,15/49,9/51		41.90 ± 9.85/43.89 ± 10.53/42.83 ± 11.27		4w	VAS, TER, HAMD
Li et al. (28)	2006	China	A + C + M	M	Once a day	33	33	11/22	13/20	38.26	39.1	2w	VAS, TER, HAMD
Liang et al. (29)	2017	China	TTN	M	Once every two days	38	38	5/33	7/31	44.1 ± 3.2	45.3 ± 3.6	2w	TER
Shao and Jiang (30)	2013	China	A	M	Once a day	18	18	11/25		39.1		8w	TER
Shi (31)	2011	China	A + C	M	Once a day	30	30	10/20	9/21	66	64.5	4w	TER
Wang et al. (32)	2002	China	A	M	Once a day	28	28	12/16	13/15	24–58	23–57	4w	TER
Wu et al. (33)	2016	China	ATET/M/ATET+M		Once a week on the seven day	33/33/33		23/76		32–65		8w	VAS, TER
Wu and Liu (34)	2022	China	A + EA	M	Once a day	41	41			42 ± 10	43 ± 10	4w	VAS, TER, HAMD
Yang et al. (35)	2015	China	A	M	Once every two days	25	25	5/20	8/17	43.3 ± 3.6	42.3 ± 4.2	6w	TER, HAMD
Yang and Liu (36)	2017	China	EA + T	M	Once a day	20	20	3/17	4/16	46.5 ± 10.19	46.20 ± 9.87	4w	TER
Yu et al. (37)	2016	China	A + TCM	M	Once a day	41	41	4/37	7/34	43.93 ± 8.08	46.88 ± 8.61	6w	VAS, TER, HAMD
Hadianfard and Hosseinzadeh Pariz (38)	2012	Iran	A	M	Once every two days	15	15	0/15	0/15	43.86 ± 7.9	44.2 ± 10.8	8w	VAS
Targino et al. (39)	2008	Brazil	A + M	M	Once every three days	34	24	0/34	0/24	52.09 ± 10.97	51.17 ± 11.20	12w	VAS
Zhao and Zhu (40)	2009	China	MSM + M	M	Once a day	30	30	9/21	11/19	39.34 ± 8.24	38.6 ± 10.58	4w	VAS, TER, HAMD
Wei et al. (41)	2006	China	AI	A	Once a day	50	44	6/44	4/40	37.25 ± 8.94	36.22 ± 8.13	24w	TER
Chen et al. (42)	2010	China	FSN + C	M	Once every two days	40	40	7/33	6/34	22 ~ 51	23 ~ 52	2w	TER
Fan and Su (43)	2012	China	FSN + TM	M	Once a week on the seven day	29	27	4/25	4/23	21 ~ 50	22 ~ 51	2w	VAS, TER
Jia (44)	2013	China	EA + C + TCM	M	Once a day	72	72			58 ± 5.5		3w	VAS, TER
Gong and Hayinulla (45)	2016	China	SNK + M	M	Once a week on the seven day	60	60	34/86		38.6 ± 4.2		3w	TER
Chi (46)	2014	China	FN	M	Once every three days	104	92	24/80	11/81	42.3	41.2	4w	TER
Chen and Yao (47)	2014	China	SNK + M	M	Once a week on the seven day	30	30	4/26	7/23	37 ± 5.9	37 ± 7	4w	VAS, TER
Deluze et al. (48)	1992	Sweden	EA	A	Once every three days	36	34	3/33	13/21	46.8 ± 2.3	49 ± 2		VAS

M, man, WM, woman, I, intervention group; C, control group, A, acupuncture; C, cupping; M, medicine; ATET, acupoint thread embedding therapy; TTN, thumb tack needle; MSM, medicine-separated moxibustion; T, tuina; AI, acupoint injection; FSN, Fu’s Subcutaneous needling; TM, thermosensitive moxibustion; SNK, small needle-knife; FN, fire needle; EA, electropuncture; TCM, traditional Chinese medicine.

### 3.3 Assessment of publication bias

**Figure 2:**
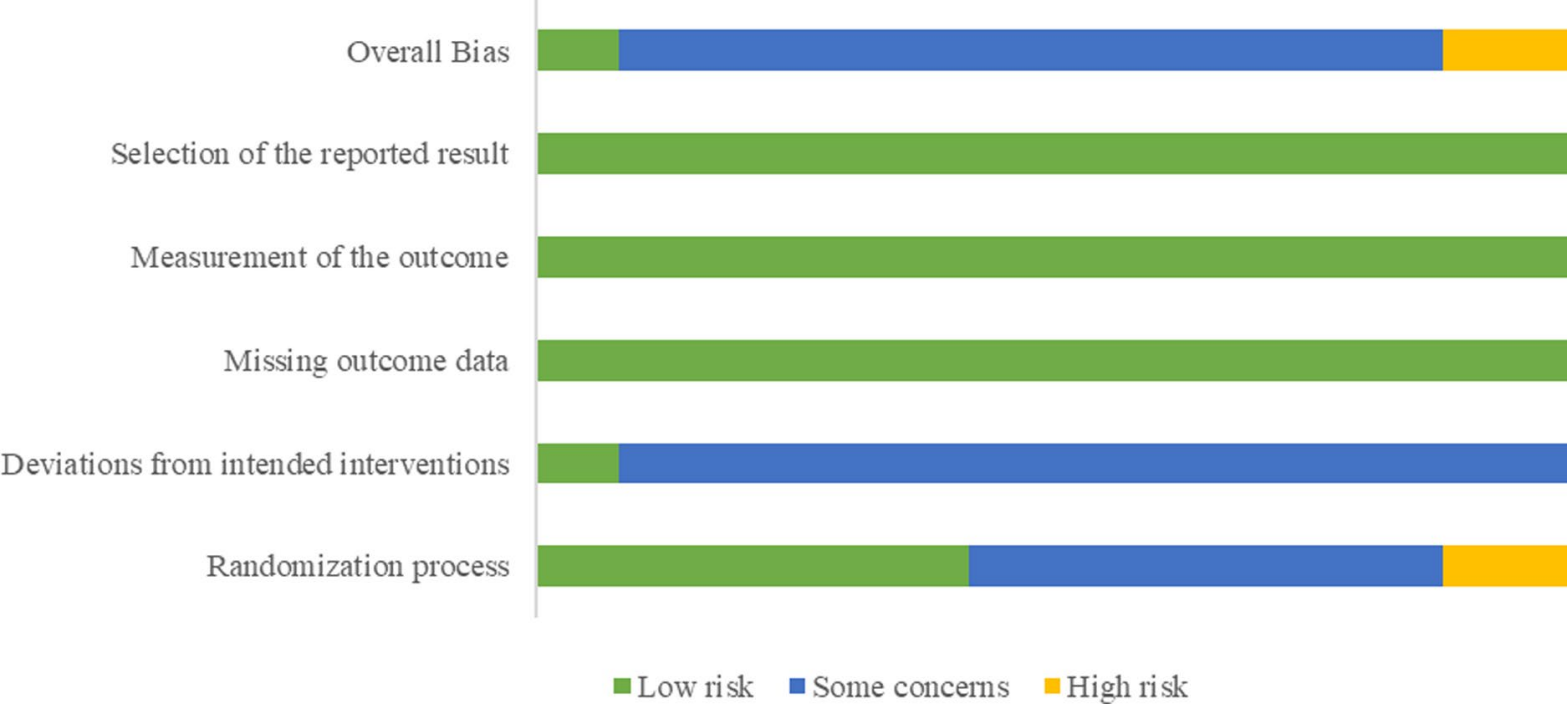
Risk of bias assessment summary (ROB2 tool).

The risk of bias assessment using the ROB2 tool showed that 3 studies (11.5%) were rated as low risk, 19 studies (73.1%) as some concerns, and 4 studies (15.4%) as high risk. The three low-risk studies were all English-language RCTs with adequate randomization sequence generation, allocation concealment, and blinding implementation, along with complete data reporting. The 19 studies rated as having some concerns had clear randomization methods and complete outcome data, but lacked blinding of operators and participants due to the specific nature of acupuncture interventions—a common limitation of acupuncture RCTs. Among the 4 high-risk studies, 3 used allocation by order of visit (inadequate randomization methods), and 1 did not describe allocation concealment or blinding implementation. All studies were rated as low risk for completeness of outcome data and selective reporting, with no evidence of significant selective reporting bias (Figure 2).

### 3.4 Meta results

#### 3.4.1 Pairwise comparison of Meta results

1. VAS

**Figure 3:**
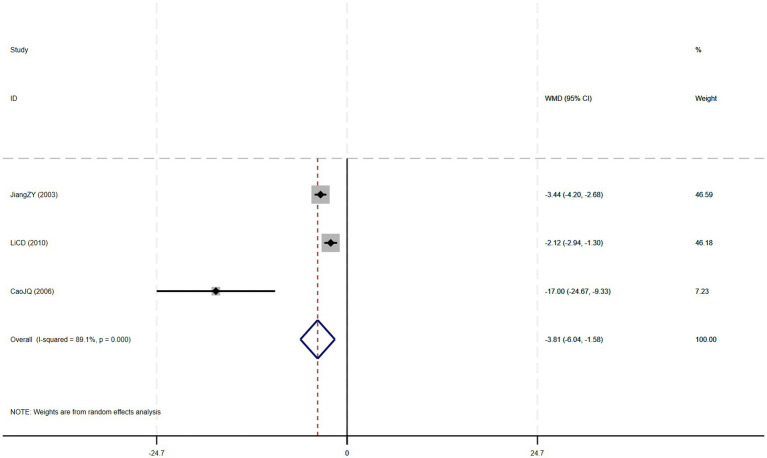
Forest plot of pairwise comparisons (VAS).

Three studies reported VAS scores, with high heterogeneity among the studies (I^2^ = 89.1%, *p* < 0.001), so a random-effects model was used to combine effect sizes. The results showed that the VAS scores in the A and C and M groups were lower than those in the M group, with statistically significant differences (WMD = −3.81, 95% CI = −6.04, −1.58) (Figure 3).

1. HAMD

**Figure 4:**
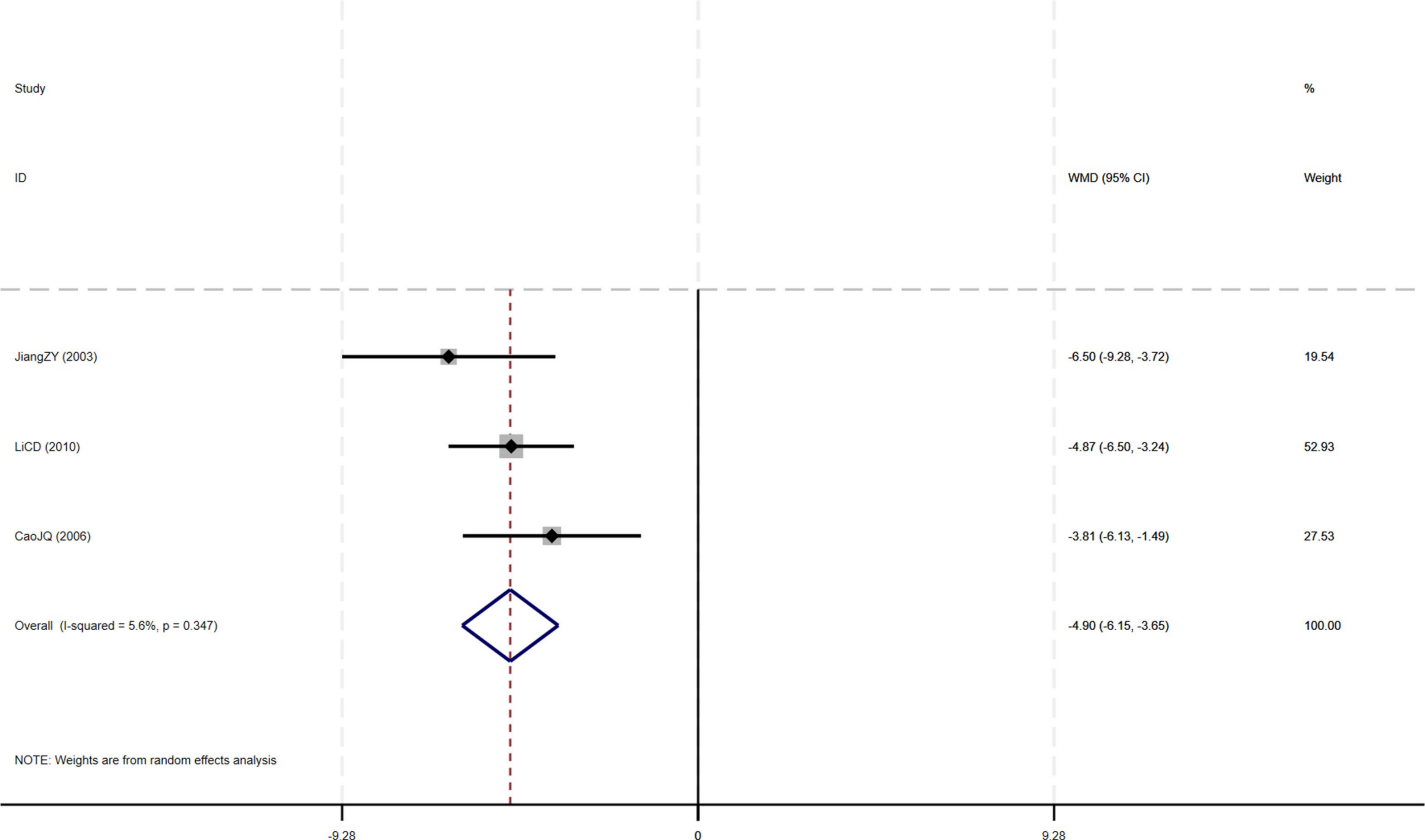
Forest plot of pairwise comparisons (HAMD).

Three studies reported HAMD scores, with low heterogeneity among studies (I^2^ = 5.6%, *p* = 0.347), so a fixed-effects model was used to combine effect sizes. The results indicated that the HAMD scores in the A and C and M group were significantly lower than those in the M group, and the difference was statistically significant (WMD = −4.90, 95% CI = −6.15 to −3.65) (Figure 4).

1. TER

**Figure 5:**
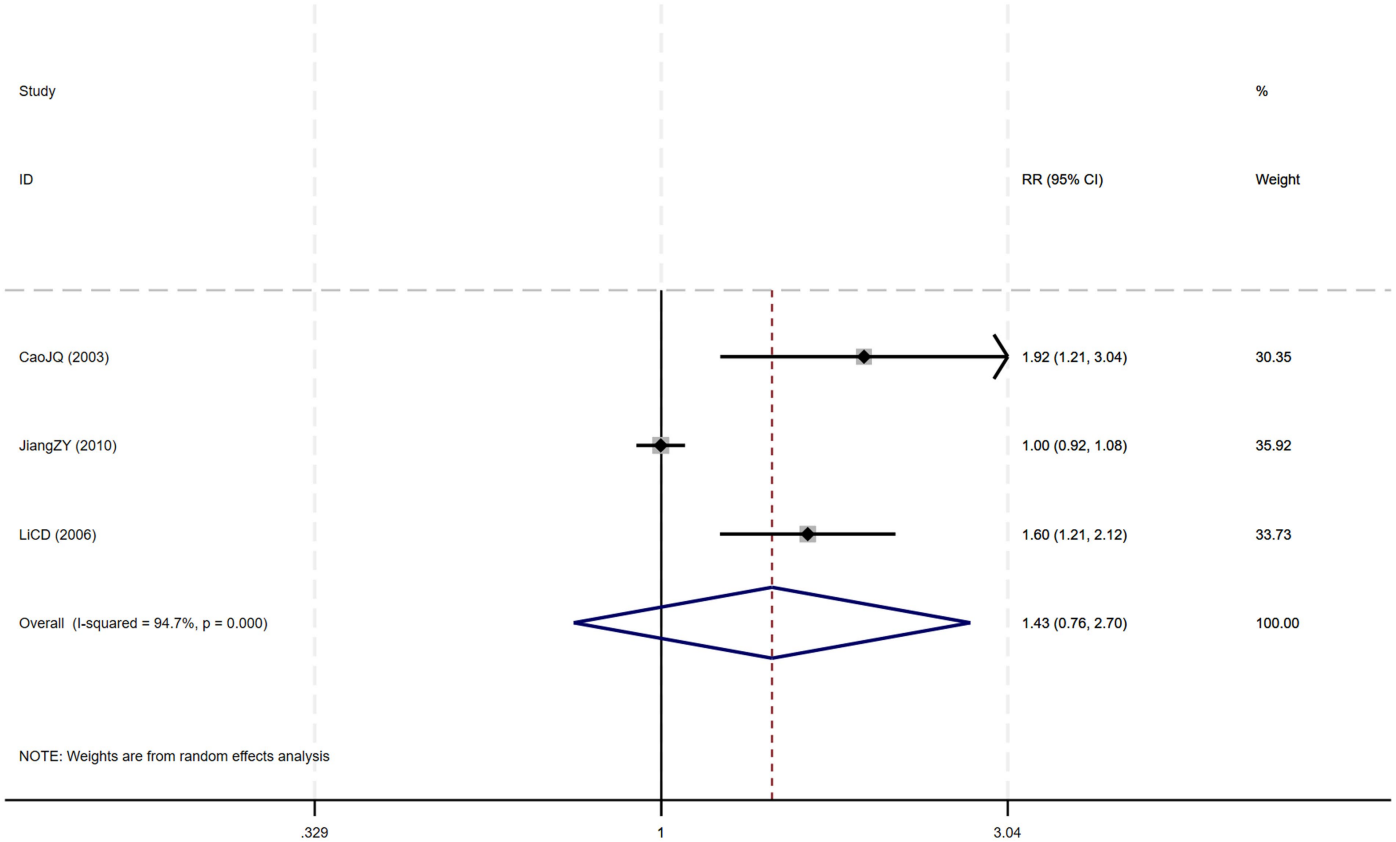
Forest plot of pairwise comparisons (TER).

Three studies reported the total effective rate, with high heterogeneity among studies (I^2^ = 94.7%, *p* < 0.001), so a random-effects model was used to combine effect sizes. The meta-analysis showed that the total effective rate in the AandCandM group was higher than in the M group, but the difference was not statistically significant (RR = 1.43, 95% CI = 0.76–2.70) (Figure 5).

#### 3.4.2 Network meta-analysis results

1. VAS

**Figure 6:**
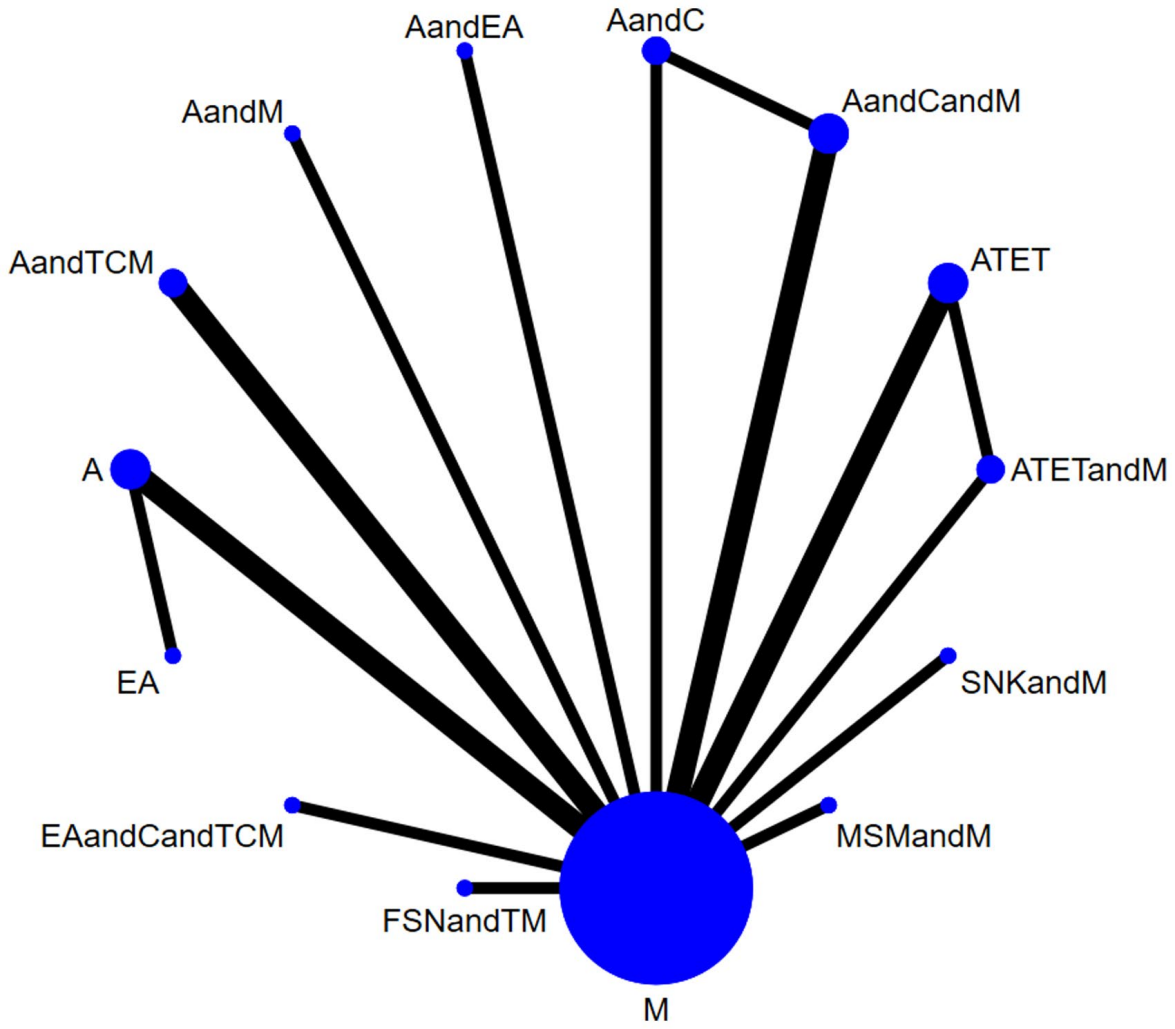
Network graph for Visual Analogue Scale (VAS).

**Figure 7:**
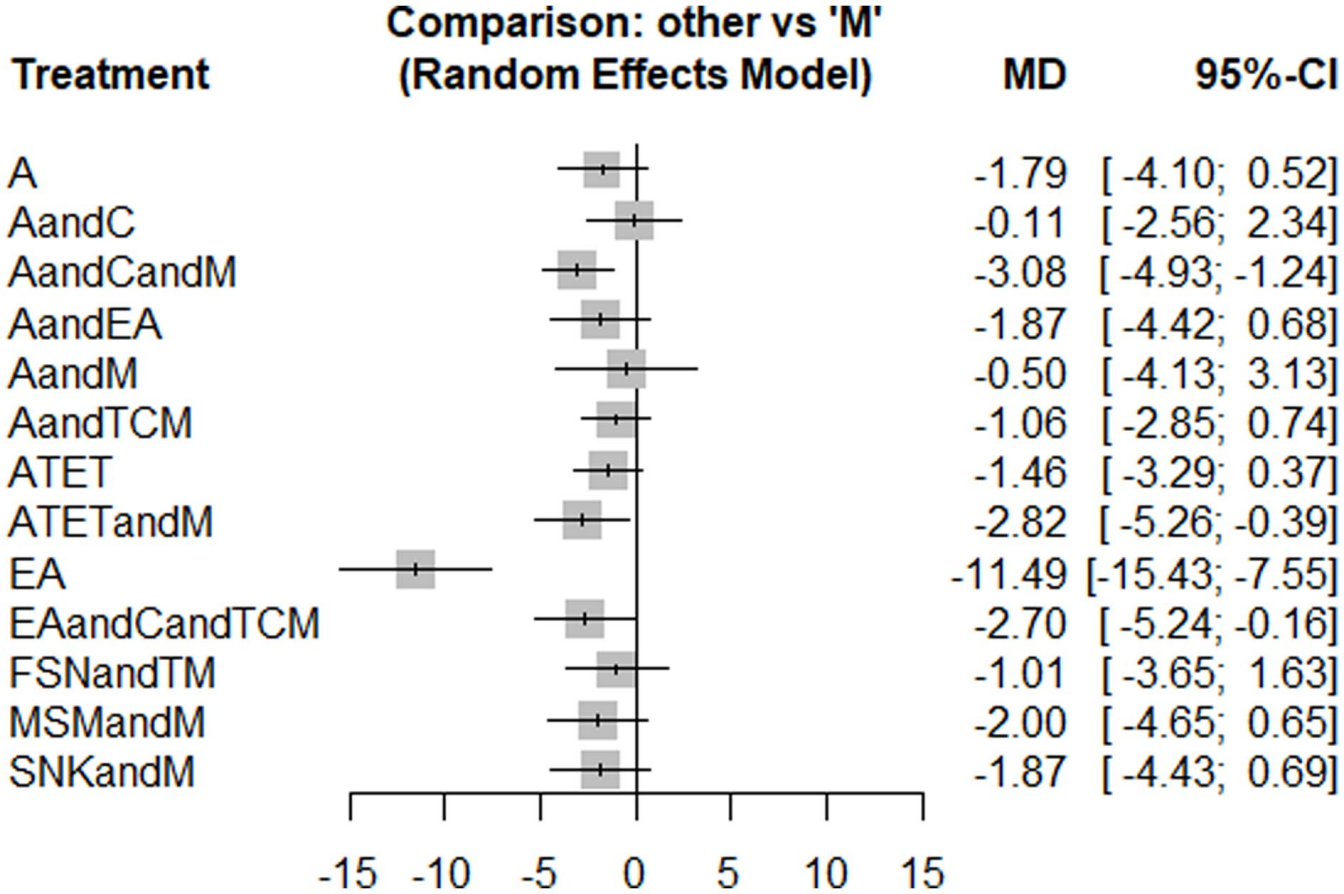
Forest plot of network meta-analysis for VAS.

Fifteen studies reported on VAS, containing 14 interventions. Among them, ATET had the most studies comparing directly with M, and the graph showed closed loops (Figure 6). Using inconsistency analysis, the results showed no statistically significant difference in the results (*p* > 0.05), using the consistency model. The network meta-analysis showed that AandCandM, ATETandM, EA, and EAandCandTCM were statistically different from drugs in terms of VAS improvement (*p* < 0.05) (Figure 7). The top three SUCRA rankings were: EA (0.99), AandCandM (0.75), and ATETandM (0.70) (Table 2). There were differences in the SUCRA rankings among acupuncture methods; however, it should be emphasized that SUCRA is a probabilistic ranking indicator and does not equate to definitive clinical superiority or inferiority. CINeMA rated this as very low-quality evidence; therefore, the ranking result should be interpreted with caution (Supplementary materials).

1. HAMD

**Table 2:** SUCRA ranking.

Intervening measure	VAS			HAMD			TER		
	SUCRA	Rank	MD (95% CI)	SUCRA	Rank	MD (95% CI)	SUCRA	Rank	RR (95% CI)
A	0.50	7	−1.79 (−4.10, 0.52)	0.65	2	−5.10 (−7.39, −2.81)	0.47	11	1.32 (1.02, 1.69)
AandC	0.19	12	−0.11 (−2.56, 2.34)	0.04	7	0.91 (−1.90, 3.72)	0.46	12	1.20 (0.88, 1.67)
AandCandM	0.75	2	−3.08 (−4.93, −1.24)	0.55	4	−4.40 (−6.34, −2.46)	0.55	8	1.35 (1.02, 1.79)
AandEA	0.52	6	−1.87 (−4.42, 0.68)	0.99	1	−10.13 (−13.90, −6.36)	0.48	10	1.32 (0.84, 2.08)
AandTCM			−1.06 (−2.85, 0.74)	0.49	5	−3.88(−6.78, −0.98)	0.73	3	1.67 (0.95, 2.94)
AI							0.30	15	1.12 (0.68, 1.85)
ATET	0.43	8	−1.46 (−3.29, 0.37)				0.52	9	1.35 (0.95, 1.92)
ATETandM	0.70	3	−2.82 (−5.26, −0.39)				0.61	6	1.35 (0.85, 2.13)
EAandCandTCM	0.67	4	−2.70 (−5.24, −0.16)				0.62	4	1.47 (0.96, 2.27)
EA and T							0.56	7	1.41 (0.81, 2.50)
FN							0.38	13	1.22 (0.81, 1.85)
FSNandC							0.74	1	1.64 (1.02, 2.70)
FSNandTM	0.35	10	−1.01 (−3.65, 1.63)				0.20	16	1.04 (0.69, 1.59)
M	0.12	13	0	0.12	6	0	0.10	17	1
MSMandM	0.54	5	−2.00 (−4.65, 0.65)	0.63	3	−5.01 (−8.61, −1.69)	0.73	2	1.64 (0.99, 2.78)
SNK and M	0.52	7	−1.87 (−4.43, 0.69)				0.61	5	1.45 (1.05, 2.00)
TTN							0.36	14	1.20 (0.78, 1.85)
AandM	0.29	11	−0.50 (−4.13, 3.13)						
AandTCM	0.35	9							
EA	0.99	1	−11.49 (−15.43, −7.55)						

A, acupuncture; C, cupping; M, medicine; ATET, acupoint thread embedding therapy; TTN, thumb tack needle; MSM, medicine-separated moxibustion; T, tuina; AI, acupoint injection; FSN, Fu’s Subcutaneous needling; TM, thermosensitive moxibustion; SNK, small needle-knife; FN, fire needle; EA, electropuncture; TCM, traditional Chinese medicine.

**Figure 8:**
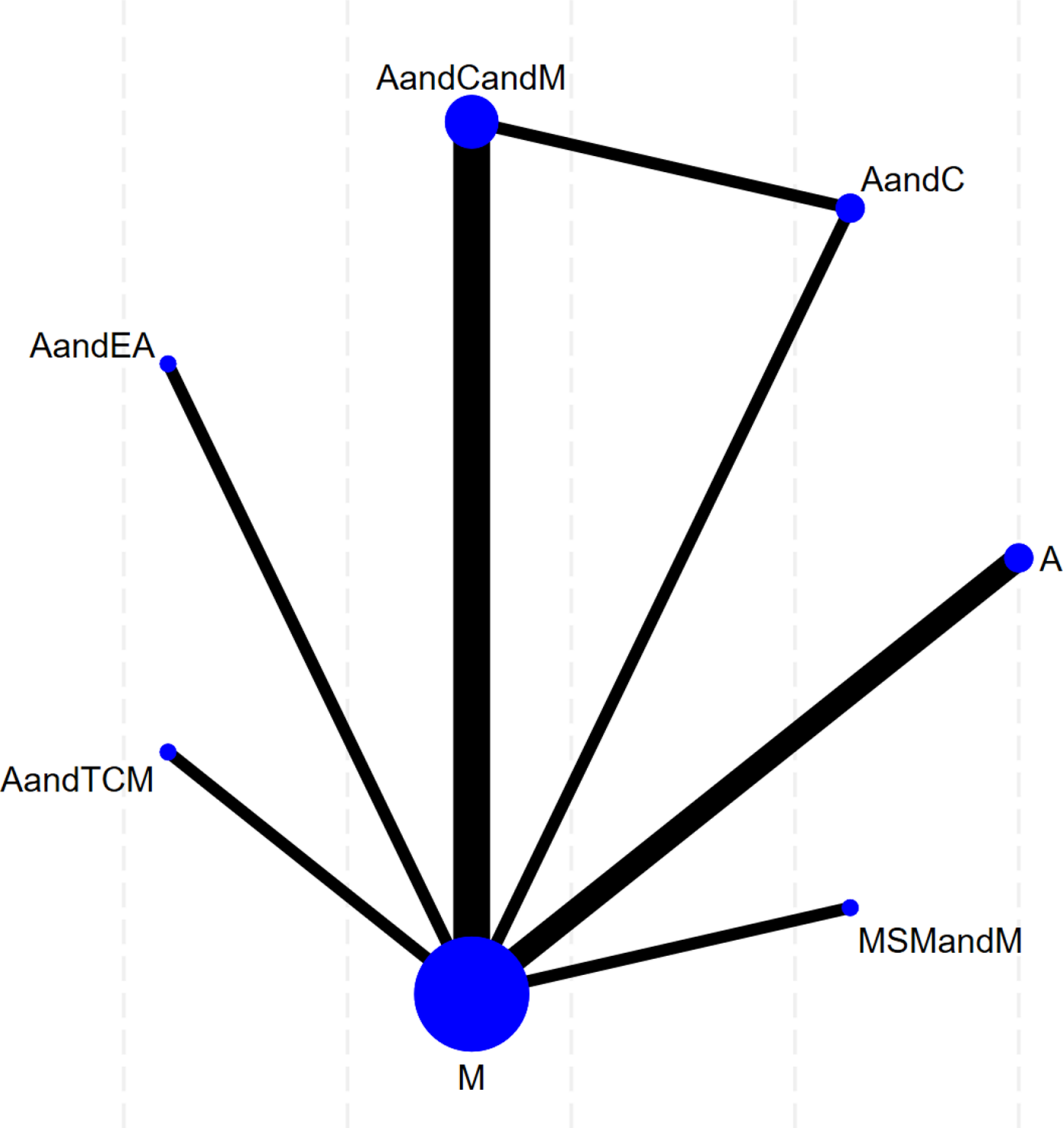
Network graph for Total Effective Rate (HAMD).

**Figure 9:**
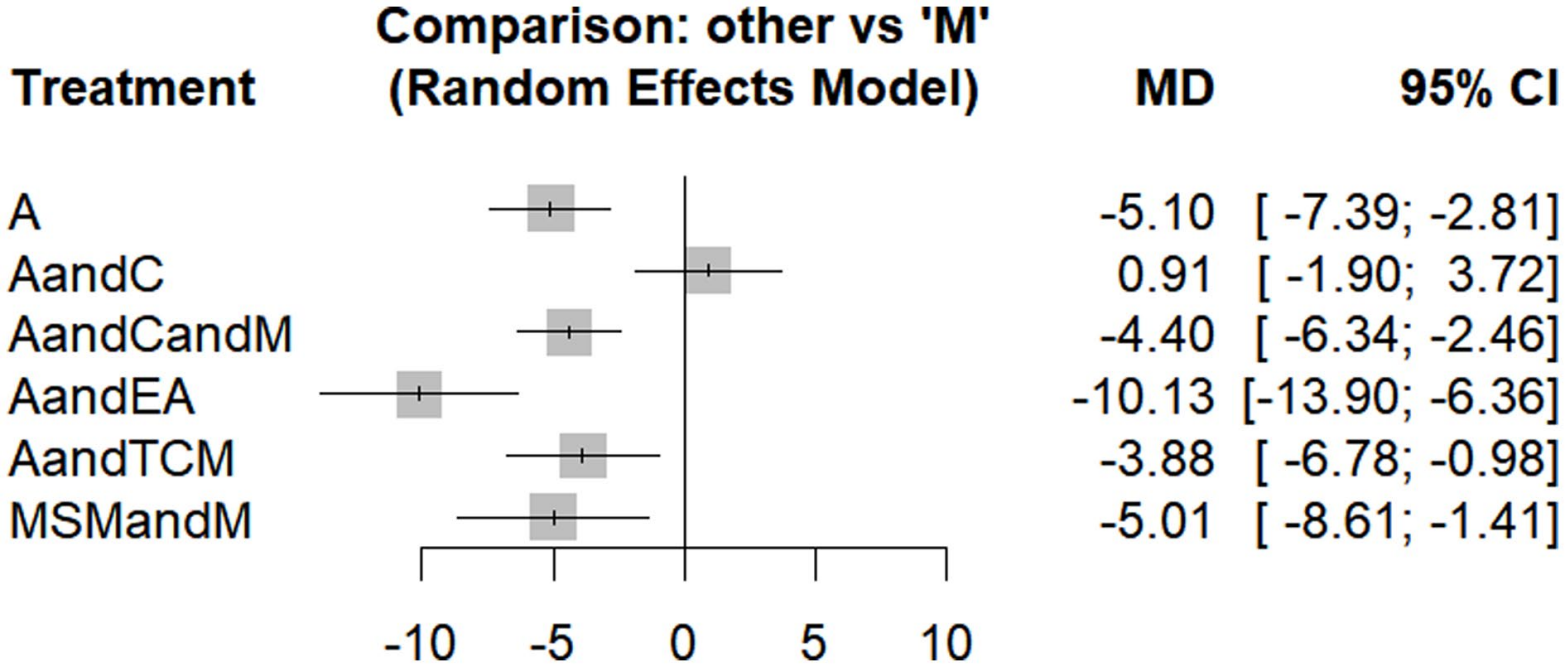
Forest plot of network meta-analysis for HAMD.

Eight studies reported HAMD, involving six interventions. Among them, the number of direct comparisons between A and C and M was the highest, shown as a closed loop in the graph (Figure 8). Inconsistency analysis revealed no statistically significant differences in results (*p* > 0.05), using the consistency model. The network meta-analysis results showed that A, A and C and M, and A and E had statistically significant differences compared to medication in improving HAMD scores (*p* < 0.05) (Figure 9). The top three SUCRA rankings were: A and E (0.94), A (0.64), and MSM and M (0.61) (Table 2). There were differences in SUCRA rankings among the acupuncture methods; however, SUCRA is merely a probabilistic ranking indicator and should be interpreted in combination with effect size, confidence intervals, and evidence quality, and does not represent definitive clinical superiority (Supplementary material).

1. TER

**Figure 10:**
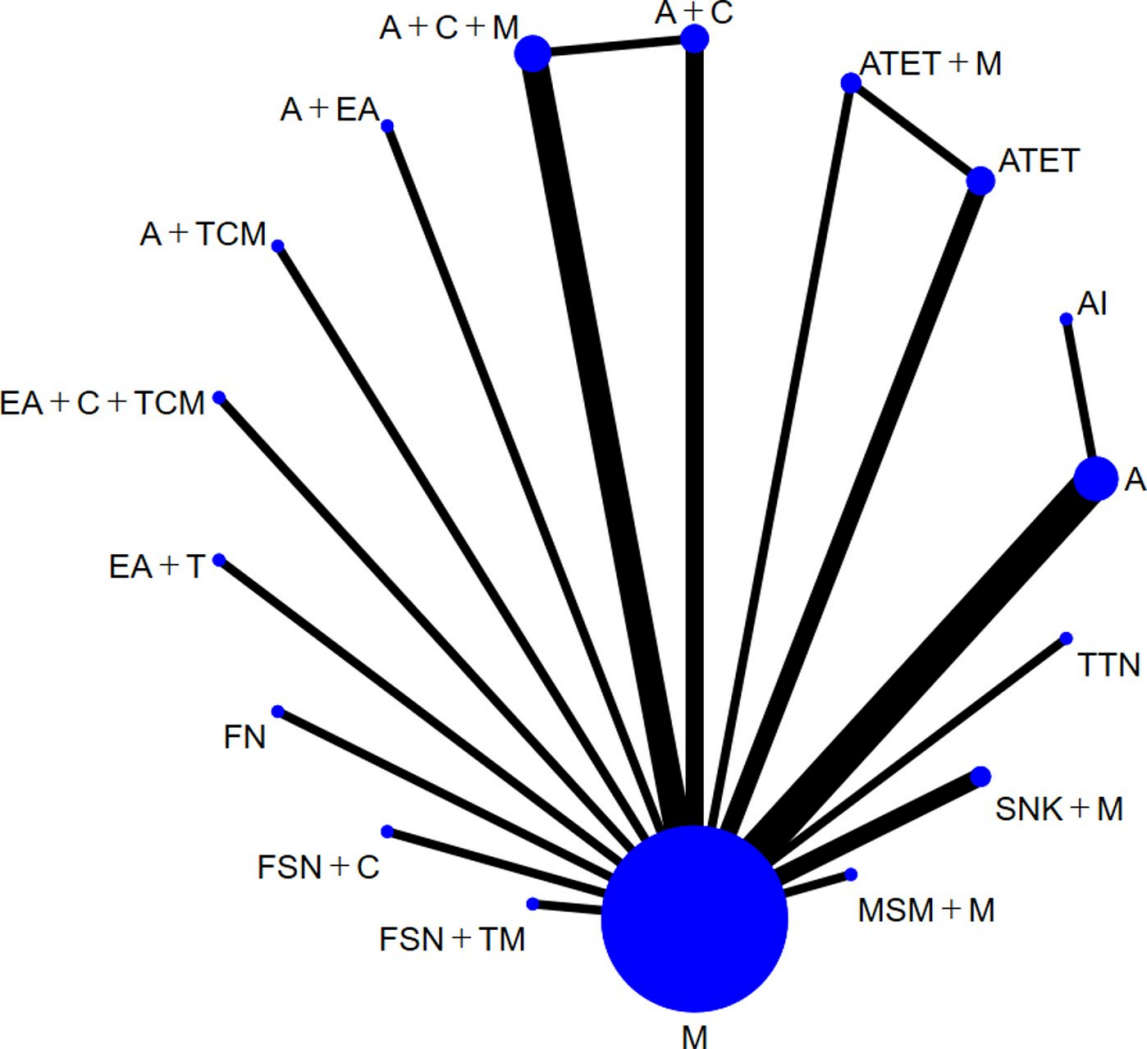
Network graph for Hamilton Depression Rating Scale (TER).

**Figure 11:**
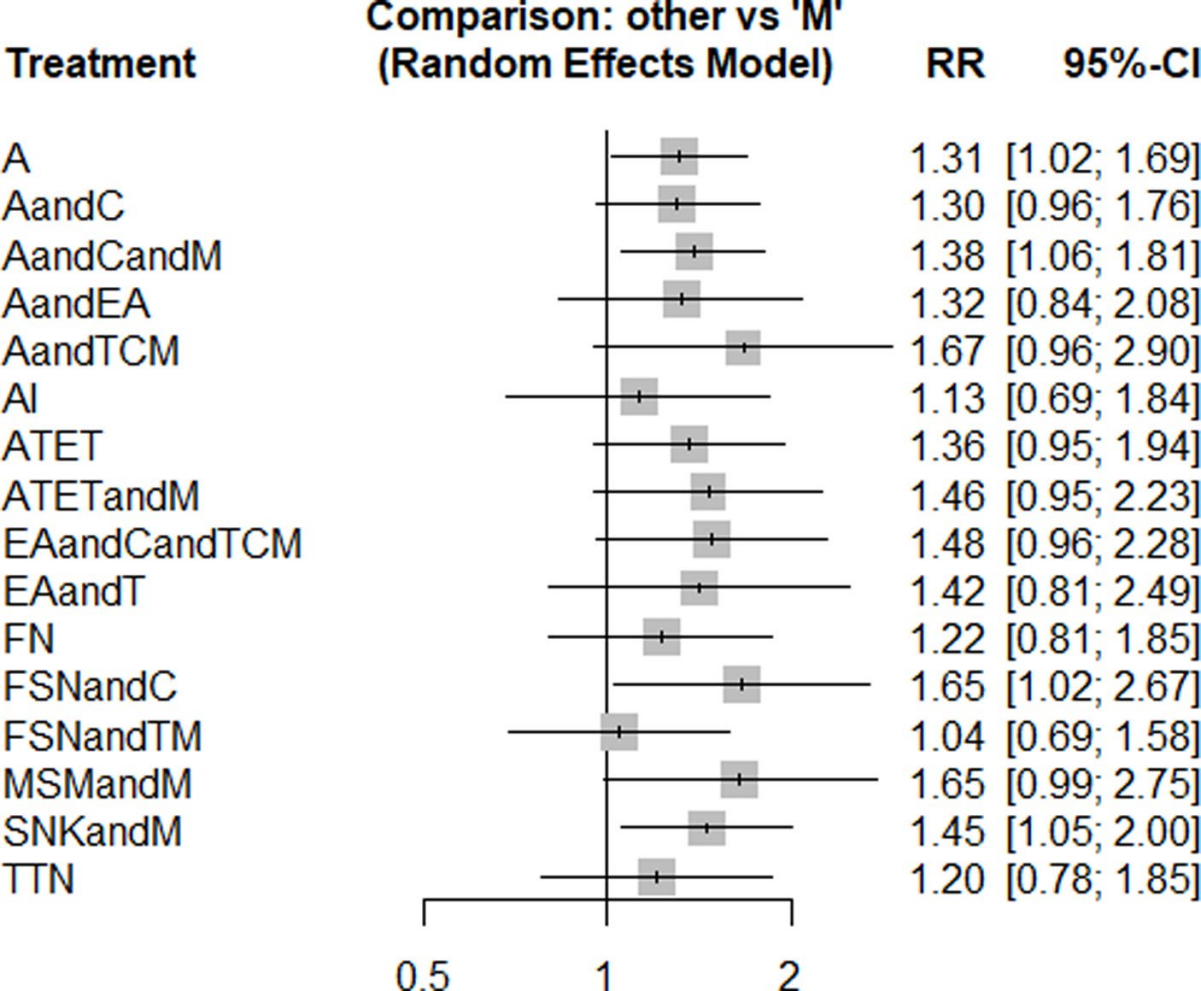
Forest plot of network meta-analysis for TER.

TER was reported in 22 studies with 17 interventions. Among them, the direct comparison between A and C had the most studies, and the graph showed closed loops (Figure 10). Using inconsistency analysis, the results showed that the results were not statistically different (*p* > 0.05), and the consistency model was used. The results of the network meta-analysis showed that A, AandCandM, FSNandC, and SNKandM had significant advantages compared with drugs (Figure 11). There were no significant differences between the acupuncture methods (Supplementary material). The top three in the SUCRA ranking were: FSNandC (0.74), MSMandM (0.73), and AandTCM (0.73) (Table 2). SUCRA is a probabilistic ranking metric and does not equate to definitive clinical superiority or inferiority; the ranking results should be interpreted with caution (Supplementary materials).

### 3.5 Sensitivity analysis and meta-regression

Sensitivity analysis of the results showed that the results of the meta-analysis were stable (Supplementary material). As the included acupuncture methods had different frequencies and treatment courses, meta-regression was performed on frequency and treatment courses to discuss their effects on the efficacy of acupuncture. The results showed that in terms of improving the effective rate, acupuncture was significantly correlated with the duration of treatment compared with drugs, showing a direct proportional relationship (Table 3).

**Table 3:** Meta-regression results.

Outcome indicator	Intervening measure	Frequency (RR/MD (95% CI))	Time (RR/MD (95% CI))
TER	A.AI	−1.11 (−8.73, 25.50)	−2.41 (−47.49, 45.34)
	A.M	−1.38 (−2.98, 0.07)	**−1.73 (−3.11, -0.48)**
	M.AandC	1.58 (−17.24, 20.95)	1.46 (−2.27, 4.83)
	M.AandCandM	1.86 (0.46, 3.37)	1.33 (−1.29, 3.20)
	M.AandEA	1.55 (−6.61, 18.23)	1.47 (−10.72, 4.90)
	M.AandTCM	1.25 (−7.53, 10.37)	1.24 (−2.32, 3.52)
	M.ATET	0.57 (−23.21, 8.63)	1.33 (−0.07, 2.78)
	M.ATETandM	2.23 (−16.65, 14.81)	2.20 (−5.55, 8.44)
	M.EAandCandTCM	1.85 (−41.26, 13.52)	2.33 (−17.74, 7.21)
	M.EAandT	1.68 (−5.17, 21.25)	1.40 (−3.79, 5.47)
	M.FN	1.25 (−4.76, 20.39)	1.22 (−3.17, 4.94)
	M.FSNandC	2.76 (−4.29, 7.49)	2.99 (−3.99, 14.49)
	M.FSNandTM	1.09 (−12.06, 14.32)	1.07 (−5.98, 8.93)
	M.MSMandM	2.59 (−8.78, 13.28)	2.60 (−1.23, 6.87)
	M.SNKandM	3.09 (−9.46, 47.72)	2.92 (0.22, 6.14)
	M.TTN	1.79 (−3.35, 7.82)	1.67 (−5.54, 7.54)
VAS	A.EA	−9.50 (−44.34, 11.22)	−9.65 (−34.65, 4.38)
	A.M	1.49 (−3.04, 6.02)	0.72 (−8.74, 9.62)
	M.AandC	−1.69 (−44.75, 16.17)	−0.09 (−10.66, 28.81)
	M.AandCandM	−3.28 (−18.96, 16.41)	−4.95 (−11.51, 4.00)
	M.AandEA	−2.85 (−30.79, 16.02)	−2.07 (−11.83, 6.75)
	M.AandM	−0.08 (−37.13, 48.62)	1.76 (−39.01, 85.47)
	M.AandTCM	−4.46 (−67.77, 13.55)	−1.42 (−5.72, 3.11)
	M.ATET	0.28 (−13.26, 27.83)	−1.63 (−5.94, 2.86)
	M.ATETandM	−4.00 (−37.79, 59.46)	−1.98 (−16.64, 24.85)
	M.EAandCandTCM	−5.23 (−75.79, 21.20)	−2.38 (−15.17, 19.89)
	M.FSNandTM	−2.21 (−23.25, 37.07)	3.01 (−54.371, 97.81)
	M.MSMandM	−2.44 (−29.02, 15.36)	−1.65 (−14.21, 37.70)
	M.SNKandM	−3.83 (−36.13, 16.00)	−1.98 (−20.39, 10.04)
HAMD	M.A	−4.40 (−10.47, 1.46)	−6.42 (−14.08, 2.32)
	M.AandC	1.60 (−16.87, 26.35)	0.76 (−16.00, 21.83)
	M.AandCandM	−4.24 (−9.09, 0.34)	−4.89 (−12.15, 2.08)
	M.AandEA	−9.96 (−34.42, 52.06)	−10.23 (−88.61, 22.90)
	M.AandTCM	−3.71 (−33.50, 13.73)	−3.89 (−16.45, 7.74)
	M.MSMandM	−5.05 (−24.48, 11.60)	−4.67 (−23.43, 70.45)

A, acupuncture; C, cupping; M, medicine; ATET, acupoint thread embedding therapy; TTN, thumb tack needle; MSM, medicine-separated moxibustion; T, tuina; AI, acupoint injection; FSN=Fu’s Subcutaneous needling; TM, thermosensitive moxibustion; SNK, small needle-knife; FN, fire needle; EA, electropuncture; TCM, traditional Chinese medicine; Red, statistically significant.

### 3.6 Publication bias analysis

**Figure 12:**
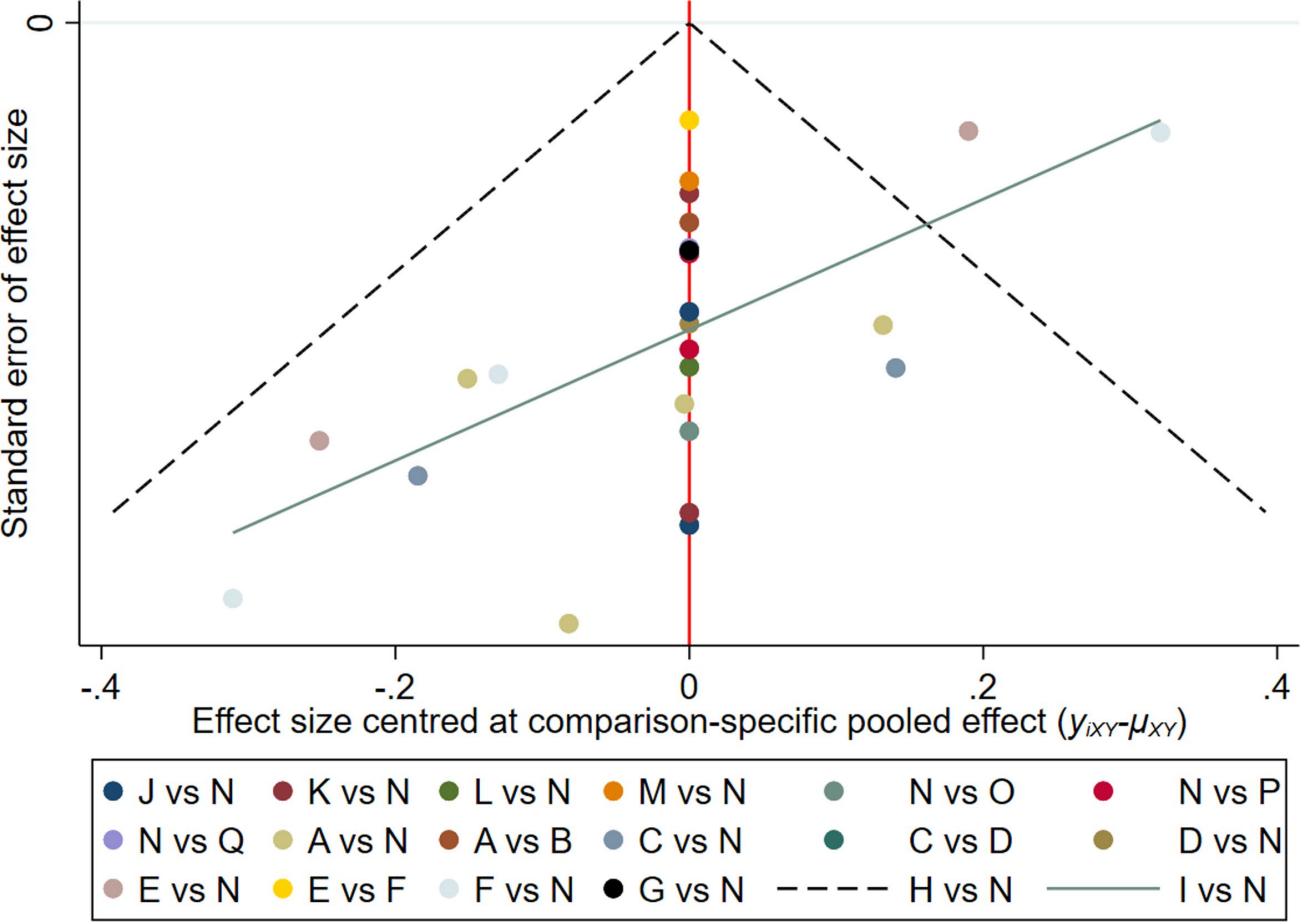
Funnel plot for publication bias assessment (TER).

**Figure 13:**
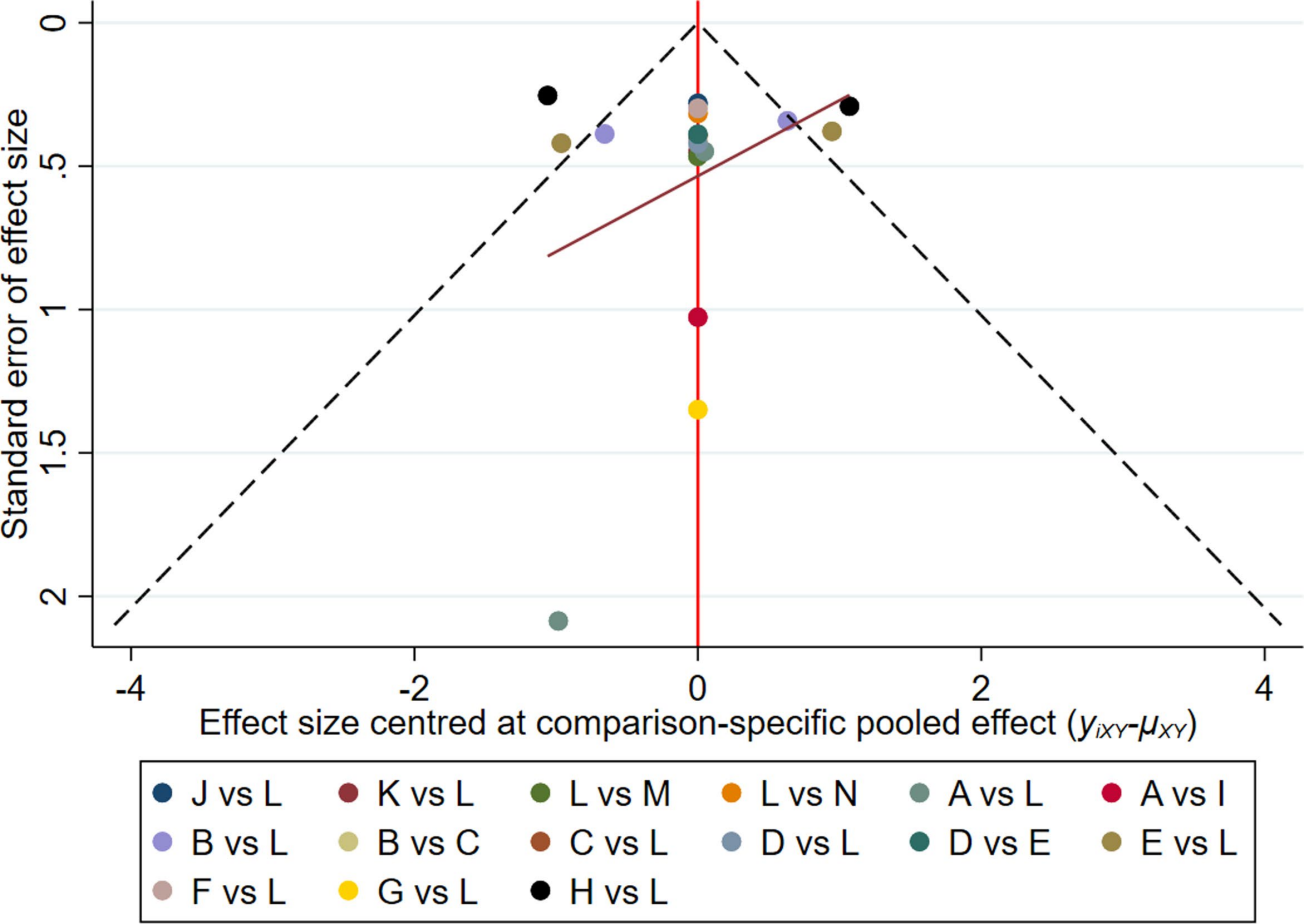
Funnel plot for publication bias assessment (VAS).

Separate comparative-adjusted funnel plots were performed for the two primary outcome measures (VAS and TER). The results showed that the study sites were distributed roughly symmetrically on both sides of the effect estimates, and no significant asymmetric patterns were observed. According to the qualitative evaluation framework recommended by the Cochrane handbook, no significant publication bias or small sample effect was found. However, it should be pointed out that due to the limited number of studies in each comparison in the network structure, publication bias may still exist, and the information conveyed by the symmetry of the funnel plot should be interpreted with caution (Figures 12, 13).

### 3.7 Quality of evidence assessment

There were 18 pieces of evidence for TER, with 10 (55.6%) of low quality and 8 (44.4%) of very low quality; 16 pieces of evidence for VAS, with 13 (81.2%) of low quality and 3 (18.8%) of very low quality; and 6 pieces of evidence for HAMD, with 5 (83.3%) of low quality and 1 (16.7%) of very low quality. Among all 40 pieces of evidence, none were high or moderate quality, while 28 (70.0%) were low quality and 12 (30.0%) were very low quality, indicating overall evidence quality ranged from low to very low. The main reasons for downgrading were imprecision (most comparisons based on only 1–2 small-sample studies with wide confidence intervals), followed by heterogeneity and inconsistency. These findings suggest that the conclusions of this study should be interpreted cautiously, and future large-scale, multicenter, high-quality randomized controlled trials are needed to enhance the credibility of the evidence base (Supplementary material).

## 4 Discussion

This study systematically compared the clinical effectiveness of different acupuncture therapies for treating fibromyalgia. Pairwise meta-analyses indicated that acupuncture combined with cupping and medication was superior to medication alone in improving pain and depression. In the network meta-analysis, based on pain improvement (measured by VAS scores)—an internationally recognized core outcome measure—electroacupuncture ranked highest in terms of probability of efficacy; however, this finding was based solely on one small-sample study (*n* = 36), and the evidence quality was rated as very low according to CINeMA. Regarding TER (a non-standardized exploratory composite outcome), floating needle combined with cupping ranked highest. Meta-regression suggested a positive correlation between acupuncture efficacy and treatment duration. It should be emphasized that SUCRA reflects probabilistic ranking rather than absolute clinical superiority, and overall evidence quality ranged from low to very low (70.0% low quality, 30.0% very low quality), primarily due to imprecision, heterogeneity, and inconsistency. Therefore, all findings should be regarded as exploratory rather than definitive conclusions. The use of TER as a composite outcome has not been standardized internationally in fibromyalgia research; different studies employ varying criteria for defining “effectiveness,” and there is no consensus on component weighting, making cross-study comparisons challenging. Future research should prioritize the adoption of the six core outcome domains recommended by OMERACT (including pain, fatigue, sleep, physical function, depression, and quality of life), and conduct more methodologically rigorous, international multicenter, large-scale randomized controlled trials with long-term follow-up to enhance the credibility and global acceptance of the evidence base.

Fu’s acupuncture therapy is a new type of acupuncture therapy, which uses disposable Fu’s acupuncture as a treatment tool to sweep and disperse acupuncture activities in the subcutaneous tissue around local pain. The analgesic effect of Fu’s acupuncture is achieved by following the meridian sensation, the luring effect and the pathogenetic circulation. The sweeping and dispersing action of Fu’s acupuncture can expand the treatment area, which exactly corresponds to the characteristics of fibromyalgia syndrome with wide pain area, shallow disease location and mostly attached to muscles (50, 51). Cupping therapy is a relatively superficial treatment in the external treatment of traditional Chinese medicine, which uses the change of air pressure to adsorb glass or bamboo jars to the skin of the human body. The therapeutic mechanism of cupping therapy is to adjust the body function and eliminate the cause through the local and systemic reactions caused by mechanical negative pressure stimulation and warm effect, so as to achieve the purpose of treatment. This therapy regulates blood circulation, strengthens metabolism, improves the nutrient supply of tissues, enhances immunity, and regulates muscle function to relieve body pain by making local tissues hyperemic and edema (43). Fu’s subcutaneous needling combined with cupping therapy, as an innovative combination of external therapy of traditional Chinese medicine, shows a unique multi-target regulation mechanism in the treatment of fibromyalgia. Fu’s acupuncture can effectively release myofascial adhesion and improve local microcirculation disorders through superficial subcutaneous puncture. The negative pressure effect produced by cupping therapy can promote the clearance of inflammatory mediators, and the synergistic effect of the two can significantly alleviate the characteristic “trigger point” symptoms of fibromyalgia. This combination therapy can not only quickly reduce the degree of pain, but also significantly improve the accompanying sleep disorders and fatigue symptoms, and its immediate analgesic effect is often reflected after the first treatment. Compared with single therapy, the combination of Fu’s subcutaneous acupuncture and cupping therapy can achieve complementary advantages, with Fu’s subcutaneous acupuncture focusing on solving structural abnormalities at the meridian and tendon level, while cupping therapy focusing on improving qi and blood circulation disorders, providing a more comprehensive intervention program for this complex disorder of fibromyalgia. However, the above mechanisms are mainly based on theoretical reasoning and limited basic research, which need to be verified by rigorously designed experiments. Compared with traditional acupuncture, Fu’s acupuncture does not penetrate deep into the muscular layer, and the incidence of adverse events (such as needle stagnation and dizzy) is low. No serious adverse events were reported in the Fu’s subcutaneous needling combined with cupping therapy group. It should be noted that the evidence for this combination therapy is mainly from single-center and small-sample studies, and the reproducibility of its efficacy needs to be further confirmed by multi-center and large-sample trials.

Electroacupuncture (EA) ranked the highest in the VAS score of SUCRA in this study (0.99). The underlying mechanism of its analgesic effect is mainly derived from preclinical studies. Animal experiments have shown that EA can down-regulate the over-expression of TRPV1 receptor in the central nervous system and inhibit the activity of pain-associated protein kinase, thereby reducing mechanical and thermal hyperalgesia (52). In the intermittent cold stress-induced fibromyalgia mouse model, EA stimulation at Zusanli (ST36) can reverse the abnormal activation of TRPV1 and its downstream signals in brain regions such as thalamus and somatosensory cortex, and its analgesic effect is equivalent to that of TRPV1 gene knockout (53). In addition, EA can inhibit the activation of microglia by up-regulating the PD-L1/PD-1 pathway, reduce the expression of Toll-like receptor 4, and reduce neuroinflammatory response (54). At the same time, EA can up-regulate the expression of CB1 receptor in the endocannabinoid system, inhibit the release of nociceptive mediators, and produce continuous analgesic effect (55). It should be emphasized that the above findings are all from preclinical animal model studies, and the exact mechanism of EA in patients with fibromyalgia needs to be confirmed by translational medicine research. At the clinical level, a network meta-analysis including 41 RCTS suggested that EA may have positive effects on pain relief, mood disorders and sleep quality (56). Individual RCTS also reported better pain improvement in the EA treatment group than in the sham laser control group (57). Ea is more effective in specific subgroups of patients, and patients with a lower sum of baseline pain time have a better response to EA treatment, which provides a predictor for individualized treatment. In the treatment of myofascial pain syndrome, the analgesic effect of electroacupuncture at motor points is better than that of traditional dry acupuncture and trigger point electroacupuncture, with an average pain score reduction of 0.98 units (58, 59). Mechanistic evidence shows that the analgesic effect of electroacupuncture may involve central and peripheral multi-target regulation, including the down-regulation of TRPV1 receptor, the activation of PD-L1/PD-1 pathway and the regulation of endocannabinoid system, etc., but the above evidence mainly comes from preclinical studies (52–55). The immediate analgesic effect of Fu’s subcutaneous acupuncture may be related to the activation of bioelectrical signal transduction network in subcutaneous connective tissue (50). Cupping can trigger multiple mechanisms such as the improvement of local blood circulation, nerve reflex and immune inflammation regulation through negative pressure stimulation (59). Moxibustion activates heat-sensitive channels and endogenous descending inhibitory pathways through thermal radiation and drug transdermal absorption (50). However, high-level human clinical evidence supporting these mechanisms is still limited.

The meta regression analysis of this study showed that there was a clear positive correlation between the duration of acupuncture treatment and the total effective rate (*p* < 0.05), suggesting that prolonging the treatment period may help to improve the efficacy of acupuncture. This finding is consistent with the dose–response pattern of acupuncture in the treatment of chronic pain. A systematic review showed that acupuncture ≥2 times a week for ≥30 min can achieve higher pain relief rates, and the efficacy can be maintained for more than 18 weeks (60). In the field of fibromyalgia, a network meta-analysis including 41 RCTS also suggested that the cumulative effect of electroacupuncture increased with treatment standardization (56). From the mechanism level, repeated acupuncture can gradually reverse the “pain memory” formed by central sensitization by regulating the activation of microglia and astrocytes in the spinal cord, which requires sufficient cumulative stimulation. However, the dose-effect relationship of acupuncture may have a characteristic of diminishing margins -- when the total number of acupuncture exceeds a certain threshold, the clinical benefits of additional treatment courses gradually taper off (61). Therefore, in clinical practice, adequate treatment courses should be arranged to maximize the efficacy, while avoiding excessive treatment, under the premise of full consideration of patient response and resource cost.

Recent network meta-analyses have demonstrated the efficacy of acupuncture-related therapies in other rheumatic diseases. Fan et al. (59) found that acupoint application, electroacupuncture combined with Western medicine ranked highest in improving pain in acute gouty arthritis. Wan et al. (62) showed that electroacupuncture combined with DMARDs was most effective in improving DAS28 scores in rheumatoid arthritis. These findings, together with our results, suggest that electroacupuncture may exert broad-spectrum analgesic effects across different rheumatic conditions. As highlighted by Zhang et al. (63), the holistic, multitarget approach of TCM is particularly suited to complex multisystem disorders like fibromyalgia, offering a complementary paradigm to conventional pharmacotherapy.

With the incidence of fibromyalgia increasing year by year, acupuncture and moxibustion therapy is gradually becoming a research hotspot in this field because of its multi-target regulation advantage and good safety. In recent years, the research of acupuncture and moxibustion in the treatment of fibromyalgia has shown three significant trends: as an innovative form of traditional acupuncture, electroacupuncture shows unique advantages in improving pain and emotional symptoms by regulating TRPV1 receptor and endocannabinoid system (60, 61, 64, 65); The research focus is shifting from simple efficacy observation to mechanism exploration, especially the central analgesic mechanism and neural plasticity changes become the frontier direction. The design of clinical research has become increasingly standardized, and the number of randomized controlled trials has increased significantly. However, the quality of existing evidence is still limited by insufficient sample size and short follow-up time. We believe that the future research directions are as follows: (1) mechanism research: the regulatory effect of acupuncture on central sensitization and neuroinflammation needs to be further elucidated by combining fMRI and molecular imaging technology. (2) In terms of clinical research, it is recommended to adopt a multi-center, large-sample randomized controlled design, pay attention to the long-term efficacy of acupuncture and moxibustion, extend the follow-up time to more than 6 months, and establish a standardized efficacy evaluation system. (3) Individualized treatment: precise acupuncture and moxibustion regimen was formulated according to the pain phenotype and gene polymorphism of patients. (4) Synergistic effect: explore the synergistic effect of acupuncture and other non-pharmacological therapies (such as cognitive behavioral therapy). (5) acupuncture-moxibustion should be included in the multidisciplinary diagnosis and treatment system of fibromyalgia, especially for patients with comorbid anxiety and depression. (6) The development of clinical guidelines needs to integrate high-quality evidence and consider the actual situation of different medical systems. (7) New acupuncture-moxibustion therapies such as electroacupuncture and Fu’s Fu’s acupuncture have significant advantages in improving quality of life indicators, which provides new ideas for the development of more optimized treatment regimens.

Additionally, this study has the following limitations: First, the unique nature of acupuncture intervention makes it difficult to achieve complete blinding between patients and practitioners, which may affect the objectivity of the results. Second, significant heterogeneity exists among studies regarding acupuncture techniques, treatment frequency, and duration; although a random-effects model was used, this may still impact the stability of the findings. This study includes simple acupuncture and complex interventions combining acupuncture with medication, cupping, or Chinese herbal medicine within the same network for node definition and comparison. Since medication, cupping, or Chinese herbal medicine may have independent therapeutic effects, the effect sizes of combined intervention nodes cannot fully disentangle the contribution of acupuncture alone. Therefore, the efficacy rankings in this study should be interpreted as reflecting the overall effects of complete intervention regimens, rather than the effects of acupuncture as a standalone therapy. Future research is recommended to employ factorial designs or component network meta-analysis to further distinguish the independent contributions and interaction effects of each treatment component. Third, most included studies had small sample sizes and short follow-up periods, resulting in insufficient assessment of long-term efficacy and safety. Fourth, the majority of included studies were published in Chinese, potentially introducing language bias. Fifth, due to the limited number of studies, subgroup analyses based on treatment frequency, duration, or control measures would result in too few studies per subgroup (mostly only 1–2), leading to inadequate statistical power. Therefore, subgroup analysis was not conducted; instead, meta-regression and sensitivity analyses were used to explore sources of heterogeneity. Results indicated a positive correlation between treatment duration and efficacy (*p* < 0.05), and the main conclusions remained robust. Sixth, diagnostic criteria varied across studies (84.6% used ACR 1990, others used 2010 or 2016 versions), which might affect population homogeneity. However, after excluding non-ACR 1990 studies in sensitivity analysis, the main conclusions remained unchanged. Seventh, this study grouped pure acupuncture with combined interventions within the same network. Since drugs, cupping, or herbal medicine in combination treatments may have independent effects, the ranking results should be interpreted as reflecting the overall effect of the complete regimen rather than the isolated effect of acupuncture alone. Sensitivity analysis excluding combined interventions still showed electroacupuncture ranked highest. Eighth, although the VAS scale range was standardized, some studies reported only change values without providing raw post-treatment data, which could introduce bias. Inconsistencies in follow-up time points across studies may also affect effect size estimation. Furthermore, network meta-analysis relies heavily on assumptions of consistency for indirect comparisons; although no significant inconsistency was detected using node-splitting methods, potential bias cannot be entirely ruled out. Finally, CINeMA evidence quality assessment revealed that overall evidence quality ranged from low to very low (70.0% low, 30.0% very low). The primary reasons for downgrading were imprecision, heterogeneity, and inconsistency, indicating that further high-quality studies are needed to validate the conclusions. Future research should employ individual patient data meta-analysis (IPD-MA) to integrate original data and adjust baseline covariates using multilevel models. Standardized reporting guidelines for acupuncture interventions should be established, clearly documenting needling parameters, practitioner qualifications, and blinding details. To address inconsistency, modular network designs are recommended, grouping similar interventions together for consistency testing, with sensitivity analyses within the framework to assess bias impact. Future studies should focus on validating the predictive value of posterior cingulate gray matter volume and conduct biomarker-based precision acupuncture clinical trials (66).

## 5 Conclusion

This study systematically compared the clinical effectiveness of different acupuncture therapies for fibromyalgia. Based on pain improvement (measured by VAS scores), a globally recognized core outcome measure, electroacupuncture demonstrated the highest efficacy ranking. Regarding the composite outcome of overall effective rate, floating needle combined with cupping therapy ranked the highest. However, the TER metric has not yet been widely adopted in international rheumatology, and SUCRA reflects probabilistic rankings rather than absolute clinical superiority; therefore, these findings should be interpreted as exploratory. Meta-regression indicated a positive correlation between acupuncture efficacy and treatment duration. It should be further noted that the lack of standardized adoption of TER as a composite outcome measure in international fibromyalgia research represents one of the main barriers to integrating traditional Chinese acupuncture studies into the global scientific community. Different studies employ varying criteria for defining “effectiveness,” and there is no consensus on the weighting of individual components, making cross-study comparisons challenging. Future research should prioritize the use of the six core outcome domains recommended by OMERACT—pain, fatigue, sleep, physical function, depression, and quality of life—to enhance international comparability and acceptance of study results. The TER-related findings in this study are intended solely for internal reference and should not serve as a basis for international comparison. Given the current low-quality evidence, these results should be regarded as exploratory rather than definitive conclusions and require further validation through higher-quality studies.

## Data Availability

The original contributions presented in the study are included in the article/Supplementary material, further inquiries can be directed to the corresponding authors.
