# Supplementary material for: Comparison of clinical efficacy of different acupuncture methods in the treatment of fibromyalgia: a network meta-analysis based on randomized controlled trials
Source: Front Neurol. 2026 Aug 5;17:1869198. doi: 10.3389/fneur.2026.1869198 (PMC13485909; doi:10.3389/fneur.2026.1869198)
Supplement: Supplementary file 1 [file Table_1.docx]

**1.Search Strategy**

(1)PubMed,Cochrane Library,Embase

| **Search number** | **Query** |
| --- | --- |
| 1 | Acupuncture OR Acupuncture Therapy OR Acupuncture, Ear OR Moxibustion[MeSH Terms] |
| 2 | Acupuncture OR Moxibustion OR Pharmacopuncture OR Acupotomy OR Acupotomies OR knife needle OR acupotome OR Acusector OR Electroacupuncture OR Acupoint* OR Fu's Subcutaneous Needling OR Moxabustion[Title/Abstract] |
| 3 | 1 OR 2 |
| 4 | Fibromyalgia[MeSH Terms] |
| 5 | Fibromyalgia OR Diffuse Myofascial Pain Syndrome OR Fibrositis OR Fibrositides OR Muscular Rheumatism[Title/Abstract] |
| 6 | 4 OR 5 |
| 7 | Randomised OR Randomized OR Blind OR phase OR multicentre OR control[All Fields] |
| 8 | 3 AND 6 AND 7 |

(2)Web of science

| **Search number** | **Query** |
| --- | --- |
| 1 | Acupuncture OR Moxibustion OR Pharmacopuncture OR Acupotomy OR Acupotomies OR knife needle OR acupotome OR Acusector OR Electroacupuncture OR Acupoint* OR Fu's Subcutaneous Needling OR Moxabustion[Topic] |
| 2 | Fibromyalgia OR Diffuse Myofascial Pain Syndrome OR Fibrositis OR Fibrositides OR Muscular Rheumatism[Topic] |
| 3 | Randomised OR Randomized OR Blind OR phase OR multicentre OR control[All Fields] |
| 4 | 1 AND 2 AND 3 |

(3)CNKI,Wanfang,VIP,SinoMed

| **Step** | **Search term** |
| --- | --- |
| #1 | Acupuncture OR Moxibustion OR Pharmacopuncture OR Acupotomy OR Acupotomies OR knife needle OR acupotome OR Acusector OR Electroacupuncture OR Acupoint* OR Fu's Subcutaneous Needling OR Moxabustion[Topic] |
| #2 | Fibromyalgia OR Diffuse Myofascial Pain Syndrome OR Fibrositis OR Fibrositides OR Muscular Rheumatism[Topic] |
| #3 | #1 AND #2 |

**2.League Table**

(1)TER

| M | FSNandTM | AI | TTN | FN | AandC | A | AandEA | ATET | AandCandM | EAandT | ATETandM | SNKandM | EAandCandTCM | MSMandM | AandTCM | FSNandC |
| --- | --- | --- | --- | --- | --- | --- | --- | --- | --- | --- | --- | --- | --- | --- | --- | --- |
| M | 0.96 (0.63, 1.45) | . | 0.83 (0.54, 1.28) | 0.82 (0.54, 1.24) | 0.83 (0.60, 1.14) | 0.76 (0.59, 0.98) | 0.76 (0.48, 1.19) | 0.74 (0.52, 1.05) | 0.74 (0.56, 0.98) | 0.71 (0.40, 1.24) | 0.74 (0.47, 1.18) | 0.69 (0.50, 0.95) | 0.68 (0.44, 1.04) | 0.61 (0.36, 1.01) | 0.60 (0.34, 1.05) | 0.61 (0.37, 0.98) |
| 0.96 (0.63, 1.45) | FSNandTM | . | . | . | . | . | . | . | . | . | . | . | . | . | . | . |
| 0.89 (0.54, 1.46) | 0.93 (0.49, 1.76) | AI | . | . | . | 0.86 (0.56, 1.31) | . | . | . | . | . | . | . | . | . | . |
| 0.83 (0.54, 1.28) | 0.87 (0.48, 1.58) | 0.94 (0.49, 1.81) | TTN | . | . | . | . | . | . | . | . | . | . | . | . | . |
| 0.82 (0.54, 1.24) | 0.85 (0.47, 1.53) | 0.92 (0.48, 1.75) | 0.98 (0.54, 1.79) | FN | . | . | . | . | . | . | . | . | . | . | . | . |
| 0.77 (0.57, 1.04) | 0.80 (0.48, 1.34) | 0.86 (0.48, 1.54) | 0.92 (0.54, 1.56) | 0.94 (0.56, 1.57) | AandC | . | . | . | 1.00 (0.67, 1.49) | . | . | . | . | . | . | . |
| 0.76 (0.59, 0.98) | 0.80 (0.49, 1.29) | 0.86 (0.56, 1.31) | 0.92 (0.55, 1.51) | 0.93 (0.57, 1.52) | 0.99 (0.67, 1.48) | A | . | . | . | . | . | . | . | . | . | . |
| 0.76 (0.48, 1.19) | 0.79 (0.43, 1.46) | 0.85 (0.43, 1.67) | 0.91 (0.48, 1.70) | 0.93 (0.50, 1.72) | 0.99 (0.57, 1.70) | 0.99 (0.59, 1.67) | AandEA | . | . | . | . | . | . | . | . | . |
| 0.74 (0.52, 1.05) | 0.77 (0.45, 1.33) | 0.83 (0.45, 1.53) | 0.88 (0.50, 1.55) | 0.90 (0.52, 1.56) | 0.96 (0.60, 1.53) | 0.97 (0.62, 1.50) | 0.97 (0.55, 1.74) | ATET | . | . | 0.87 (0.56, 1.34) | . | . | . | . | . |
| 0.72 (0.55, 0.95) | 0.75 (0.46, 1.23) | 0.81 (0.46, 1.43) | 0.87 (0.52, 1.44) | 0.88 (0.54, 1.45) | 0.94 (0.67, 1.32) | 0.95 (0.65, 1.37) | 0.96 (0.56, 1.62) | 0.98 (0.63, 1.53) | AandCandM | . | . | . | . | . | . | . |
| 0.71 (0.40, 1.24) | 0.74 (0.37, 1.48) | 0.79 (0.38, 1.68) | 0.85 (0.42, 1.72) | 0.86 (0.43, 1.74) | 0.92 (0.49, 1.74) | 0.93 (0.50, 1.71) | 0.93 (0.45, 1.92) | 0.96 (0.49, 1.87) | 0.98 (0.52, 1.82) | EAandT | . | . | . | . | . | . |
| 0.68 (0.45, 1.05) | 0.71 (0.39, 1.29) | 0.77 (0.40, 1.48) | 0.82 (0.45, 1.51) | 0.84 (0.46, 1.52) | 0.89 (0.53, 1.50) | 0.90 (0.55, 1.47) | 0.90 (0.48, 1.69) | 0.93 (0.62, 1.40) | 0.95 (0.57, 1.57) | 0.97 (0.48, 1.96) | ATETandM | . | . | . | . | . |
| 0.69 (0.50, 0.95) | 0.72 (0.43, 1.21) | 0.78 (0.43, 1.40) | 0.83 (0.48, 1.42) | 0.84 (0.50, 1.42) | 0.90 (0.58, 1.40) | 0.90 (0.60, 1.36) | 0.91 (0.52, 1.59) | 0.94 (0.58, 1.51) | 0.95 (0.63, 1.45) | 0.98 (0.51, 1.87) | 1.01 (0.59, 1.71) | SNKandM | . | . | . | . |
| 0.68 (0.44, 1.04) | 0.71 (0.39, 1.28) | 0.76 (0.39, 1.47) | 0.81 (0.44, 1.50) | 0.83 (0.45, 1.51) | 0.88 (0.52, 1.50) | 0.89 (0.54, 1.46) | 0.89 (0.48, 1.68) | 0.92 (0.52, 1.61) | 0.94 (0.56, 1.56) | 0.96 (0.47, 1.95) | 0.99 (0.54, 1.81) | 0.98 (0.57, 1.68) | EAandCandTCM | . | . | . |
| 0.61 (0.36, 1.01) | 0.63 (0.33, 1.22) | 0.68 (0.34, 1.39) | 0.73 (0.37, 1.42) | 0.74 (0.38, 1.44) | 0.79 (0.44, 1.43) | 0.80 (0.45, 1.41) | 0.80 (0.40, 1.59) | 0.82 (0.44, 1.54) | 0.84 (0.47, 1.50) | 0.86 (0.40, 1.84) | 0.89 (0.46, 1.72) | 0.88 (0.48, 1.61) | 0.90 (0.46, 1.75) | MSMandM | . | . |
| 0.60 (0.34, 1.05) | 0.63 (0.31, 1.25) | 0.68 (0.32, 1.42) | 0.72 (0.36, 1.46) | 0.73 (0.37, 1.47) | 0.78 (0.42, 1.47) | 0.79 (0.43, 1.45) | 0.79 (0.39, 1.63) | 0.81 (0.42, 1.58) | 0.83 (0.45, 1.54) | 0.85 (0.39, 1.87) | 0.88 (0.44, 1.76) | 0.87 (0.46, 1.65) | 0.89 (0.44, 1.79) | 0.99 (0.46, 2.10) | AandTCM | . |
| 0.61 (0.37, 0.98) | 0.63 (0.34, 1.19) | 0.68 (0.34, 1.36) | 0.73 (0.38, 1.39) | 0.74 (0.39, 1.40) | 0.79 (0.45, 1.39) | 0.79 (0.46, 1.36) | 0.80 (0.41, 1.55) | 0.82 (0.45, 1.49) | 0.84 (0.48, 1.45) | 0.86 (0.41, 1.80) | 0.88 (0.47, 1.68) | 0.88 (0.49, 1.56) | 0.89 (0.47, 1.71) | 1.00 (0.49, 2.01) | 1.01 (0.48, 2.10) | FSNandC |

(2)VAS

| EA | AandCandM | ATETandM | EAandCandTCM | MSMandM | AandEA | SNKandM | A | ATET | FSNandTM | AandTCM | AandM | AandC | M |
| --- | --- | --- | --- | --- | --- | --- | --- | --- | --- | --- | --- | --- | --- |
| EA | . | . | . | . | . | . | -9.70 (-12.90, -6.50) | . | . | . | . | . | . |
| -8.41 (-12.76, -4.06) | AandCandM | . | . | . | . | . | . | . | . | . | . | -3.44 ( -6.04, -0.84) | -3.08 ( -4.93, -1.24) |
| -8.67 (-13.30, -4.03) | -0.26 ( -3.31, 2.79) | ATETandM | . | . | . | . | . | -1.69 ( -4.30, 0.92) | . | . | . | . | -2.50 ( -5.10, 0.10) |
| -8.79 (-13.48, -4.10) | -0.38 ( -3.52, 2.76) | -0.12 ( -3.64, 3.40) | EAandCandTCM | . | . | . | . | . | . | . | . | . | -2.70 ( -5.24, -0.16) |
| -9.49 (-14.24, -4.74) | -1.08 ( -4.31, 2.14) | -0.82 ( -4.42, 2.77) | -0.70 ( -4.37, 2.97) | MSMandM | . | . | . | . | . | . | . | . | -2.00 ( -4.65, 0.65) |
| -9.62 (-14.32, -4.92) | -1.21 ( -4.36, 1.93) | -0.95 ( -4.48, 2.57) | -0.83 ( -4.43, 2.77) | -0.13 ( -3.81, 3.55) | AandEA | . | . | . | . | . | . | . | -1.87 ( -4.42, 0.68) |
| -9.62 (-14.32, -4.92) | -1.21 ( -4.37, 1.94) | -0.95 ( -4.49, 2.58) | -0.83 ( -4.44, 2.78) | -0.13 ( -3.81, 3.55) | 0.00 ( -3.61, 3.61) | SNKandM | . | . | . | . | . | . | -1.87 ( -4.43, 0.69) |
| -9.70 (-12.90, -6.50) | -1.29 ( -4.25, 1.66) | -1.03 ( -4.39, 2.32) | -0.91 ( -4.34, 2.52) | -0.21 ( -3.72, 3.30) | -0.08 ( -3.52, 3.36) | -0.08 ( -3.53, 3.37) | A | . | . | . | . | . | -1.79 ( -4.10, 0.52) |
| -10.03 (-14.37, -5.68) | -1.62 ( -4.22, 0.97) | -1.36 ( -3.80, 1.08) | -1.24 ( -4.37, 1.89) | -0.54 ( -3.75, 2.68) | -0.41 ( -3.55, 2.73) | -0.41 ( -3.55, 2.74) | -0.33 ( -3.27, 2.61) | ATET | . | . | . | . | -1.46 ( -3.29, 0.37) |
| -10.48 (-15.22, -5.74) | -2.07 ( -5.29, 1.14) | -1.81 ( -5.40, 1.78) | -1.69 ( -5.35, 1.97) | -0.99 ( -4.73, 2.75) | -0.86 ( -4.53, 2.81) | -0.86 ( -4.53, 2.81) | -0.78 ( -4.28, 2.72) | -0.45 ( -3.66, 2.76) | FSNandTM | . | . | . | -1.01 ( -3.65, 1.63) |
| -10.43 (-14.76, -6.10) | -2.03 ( -4.60, 0.55) | -1.76 ( -4.79, 1.26) | -1.64 ( -4.76, 1.47) | -0.94 ( -4.14, 2.26) | -0.81 ( -3.93, 2.31) | -0.81 ( -3.94, 2.32) | -0.73 ( -3.66, 2.19) | -0.40 ( -2.97, 2.16) | 0.05 ( -3.14, 3.24) | AandTCM | . | . | -1.06 ( -2.85, 0.74) |
| -10.99 (-16.35, -5.63) | -2.58 ( -6.65, 1.48) | -2.32 ( -6.69, 2.05) | -2.20 ( -6.63, 2.23) | -1.50 ( -5.99, 2.99) | -1.37 ( -5.80, 3.06) | -1.37 ( -5.81, 3.07) | -1.29 ( -5.59, 3.01) | -0.96 ( -5.02, 3.10) | -0.51 ( -4.99, 3.97) | -0.56 ( -4.61, 3.49) | AandM | . | -0.50 ( -4.13, 3.13) |
| -11.38 (-16.02, -6.74) | -2.97 ( -5.41, -0.54) | -2.71 ( -6.16, 0.74) | -2.59 ( -6.12, 0.94) | -1.89 ( -5.49, 1.71) | -1.76 ( -5.30, 1.78) | -1.76 ( -5.30, 1.78) | -1.68 ( -5.04, 1.68) | -1.35 ( -4.41, 1.70) | -0.90 ( -4.50, 2.70) | -0.95 ( -3.98, 2.09) | -0.39 ( -4.77, 3.99) | AandC | -0.59 ( -3.21, 2.03) |
| -11.49 (-15.43, -7.55) | -3.08 ( -4.93, -1.24) | -2.82 ( -5.26, -0.39) | -2.70 ( -5.24, -0.16) | -2.00 ( -4.65, 0.65) | -1.87 ( -4.42, 0.68) | -1.87 ( -4.43, 0.69) | -1.79 ( -4.10, 0.52) | -1.46 ( -3.29, 0.37) | -1.01 ( -3.65, 1.63) | -1.06 ( -2.85, 0.74) | -0.50 ( -4.13, 3.13) | -0.11 ( -2.56, 2.34) | M |

(3)HAMD

| AandEA | A | MSMandM | AandCandM | AandTCM | M | AandC |
| --- | --- | --- | --- | --- | --- | --- |
| AandEA |  |  |  |  | -10.13 (-13.90, -6.36) |  |
| -5.03 ( -9.44, -0.62) | A |  |  |  | -5.10 ( -7.39, -2.81) |  |
| -5.12 (-10.33, 0.09) | -0.09 ( -4.36, 4.18) | MSMandM |  |  | -5.01 ( -8.61, -1.41) |  |
| -5.73 ( -9.97, -1.49) | -0.70 ( -3.70, 2.29) | -0.61 ( -4.70, 3.48) | AandCandM | . | -4.40 ( -6.34, -2.46) | -4.87 ( -7.87, -1.87) |
| -6.25 (-11.00, -1.50) | -1.22 ( -4.91, 2.47) | -1.13 ( -5.75, 3.49) | -0.52 ( -4.00, 2.97) | AandTCM | -3.88 ( -6.78, -0.98) | . |
| -10.13 (-13.90, -6.36) | -5.10 ( -7.39, -2.81) | -5.01 ( -8.61, -1.41) | -4.40 ( -6.34, -2.46) | -3.88 ( -6.78, -0.98) | M | -1.38 ( -4.44, 1.68) |
| -11.04 (-15.74, -6.33) | -6.01 ( -9.63, -2.38) | -5.92 (-10.49, -1.35) | -5.31 ( -8.09, -2.52) | -4.79 ( -8.83, -0.75) | -0.91 ( -3.72, 1.90) | AandC |

**3.Sensitivity Analysis**

(1)TER

(2)VAS

(3)HAMD

**4.CINeMA result**

| **Outcomes** | **Comparison** | **Number of studies** | **Within-study bias** | **Reporting bias** | **Indirectness** | **Imprecision** | **Heterogeneity** | **Incoherence** | **Confidence rating** | **Quality level** |
| --- | --- | --- | --- | --- | --- | --- | --- | --- | --- | --- |
| TER | A:AI | 1 | No concerns | Low risk | No concerns | Major concerns | No concerns | Major concerns | High | Very low |
| TER | A:M | 4 | No concerns | Low risk | No concerns | No concerns | Major concerns | Major concerns | High | Low |
| TER | ATET:ATETandM | 1 | No concerns | Low risk | No concerns | Major concerns | No concerns | No concerns | High | Very low |
| TER | ATET:M | 2 | No concerns | Low risk | No concerns | Major concerns | No concerns | Major concerns | High | Very low |
| TER | ATETandM:M | 1 | No concerns | Low risk | No concerns | Major concerns | No concerns | No concerns | High | Very low |
| TER | AandC:AandCandM | 1 | No concerns | Low risk | No concerns | Major concerns | No concerns | No concerns | High | Very low |
| TER | AandC:M | 2 | No concerns | Low risk | No concerns | Major concerns | No concerns | No concerns | High | Very low |
| TER | AandCandM:M | 3 | No concerns | Low risk | No concerns | No concerns | Major concerns | No concerns | High | Low |
| TER | AandEA:M | 1 | No concerns | Low risk | No concerns | Major concerns | No concerns | Major concerns | High | Very low |
| TER | AandTCM:M | 1 | No concerns | Low risk | No concerns | Major concerns | No concerns | Major concerns | High | Very low |
| TER | EAandCandTCM:M | 1 | No concerns | Low risk | No concerns | Major concerns | No concerns | Major concerns | High | Very low |
| TER | EAandT:M | 1 | No concerns | Low risk | No concerns | Major concerns | No concerns | Major concerns | High | Very low |
| TER | FN:M | 1 | No concerns | Low risk | No concerns | Major concerns | No concerns | Major concerns | High | Very low |
| TER | FSNandC:M | 1 | No concerns | Low risk | No concerns | No concerns | Major concerns | Major concerns | High | Low |
| TER | FSNandTM:M | 1 | No concerns | Low risk | No concerns | Major concerns | No concerns | Major concerns | High | Very low |
| TER | M:MSMandM | 1 | No concerns | Low risk | No concerns | Major concerns | No concerns | Major concerns | High | Very low |
| TER | M:SNKandM | 2 | No concerns | Low risk | No concerns | No concerns | Major concerns | Major concerns | High | Low |
| TER | M:TTN | 1 | No concerns | Low risk | No concerns | Major concerns | No concerns | Major concerns | High | Very low |
| HAMD | A:M | 2 | No concerns | Low risk | No concerns | Some concerns | Some concerns | No concerns | High | Low |
| HAMD | AandC:AandCandM | 1 | No concerns | Low risk | No concerns | Major concerns | No concerns | No concerns | High | Very low |
| HAMD | AandC:M | 1 | No concerns | Low risk | No concerns | Major concerns | No concerns | No concerns | High | Very low |
| HAMD | AandCandM:M | 3 | No concerns | Low risk | No concerns | Major concerns | No concerns | No concerns | High | Very low |
| HAMD | AandEA:M | 1 | No concerns | Low risk | No concerns | Major concerns | No concerns | No concerns | High | Very low |
| HAMD | AandTCM:M | 1 | No concerns | Low risk | No concerns | Major concerns | No concerns | No concerns | High | Very low |
| HAMD | M:MSMandM | 1 | No concerns | Low risk | No concerns | Major concerns | No concerns | No concerns | High | Very low |
| VAS | A:EA | 1 | No concerns | Low risk | No concerns | Major concerns | No concerns | No concerns | High | Very low |
| VAS | A:M | 2 | No concerns | Low risk | No concerns | Major concerns | No concerns | No concerns | High | Very low |
| VAS | ATET:ATETandM | 1 | No concerns | Low risk | No concerns | Some concerns | Some concerns | No concerns | High | Low |
| VAS | ATET:M | 2 | No concerns | Low risk | No concerns | Some concerns | Some concerns | No concerns | High | Low |
| VAS | ATETandM:M | 1 | No concerns | Low risk | No concerns | Some concerns | Some concerns | No concerns | High | Low |
| VAS | AandC:AandCandM | 1 | No concerns | Low risk | No concerns | Some concerns | Some concerns | No concerns | High | Low |
| VAS | AandC:M | 1 | No concerns | Low risk | No concerns | Some concerns | Some concerns | No concerns | High | Low |
| VAS | AandCandM:M | 2 | No concerns | Low risk | No concerns | Some concerns | Some concerns | No concerns | High | Low |
| VAS | AandEA:M | 1 | No concerns | Low risk | No concerns | Major concerns | No concerns | No concerns | High | Very low |
| VAS | AandM:M | 1 | No concerns | Low risk | No concerns | Major concerns | No concerns | No concerns | High | Very low |
| VAS | AandTCM:M | 2 | No concerns | Low risk | No concerns | No concerns | Major concerns | No concerns | High | Low |
| VAS | EAandCandTCM:M | 1 | No concerns | Low risk | No concerns | Major concerns | No concerns | No concerns | High | Very low |
| VAS | FSNandTM:M | 1 | No concerns | Low risk | No concerns | Major concerns | No concerns | No concerns | High | Very low |
| VAS | M:MSMandM | 1 | No concerns | Low risk | No concerns | Major concerns | No concerns | No concerns | High | Very low |
| VAS | M:SNKandM | 1 | No concerns | Low risk | No concerns | Major concerns | No concerns | No concerns | High | Very low |
